# Genomic and Transcriptomic Landscape of Tumor Clonal Evolution in Cholangiocarcinoma

**DOI:** 10.3389/fgene.2020.00195

**Published:** 2020-03-13

**Authors:** Geng Chen, Zhixiong Cai, Xiuqing Dong, Jing Zhao, Song Lin, Xi Hu, Fang-E Liu, Xiaolong Liu, Huqing Zhang

**Affiliations:** ^1^School of Life Sciences and Technology, Xi’an Jiaotong University, Xi’an, China; ^2^The United Innovation of Mengchao Hepatobiliary Technology Key Laboratory of Fujian Province, Mengchao Hepatobiliary Hospital of Fujian Medical University, Fuzhou, China; ^3^Department of Nursing, School of Medicine, Xi’an Peihua University, Xi’an, China

**Keywords:** cholangiocarcinoma, clonal evolution, sequencing, transcriptome, genome

## Abstract

Cholangiocarcinoma remained a severe threat to human health. Deciphering the genomic and/or transcriptomic profiles of tumor has been proved to be a promising strategy for exploring the mechanism of tumorigenesis and development, which could also provide valuable insights into Cholangiocarcinoma. However, little knowledge has been obtained regarding to how the alteration among different omics levels is connected. Here, using whole exome sequencing and transcriptome sequencing, we performed a thorough evaluation for the landscape of genome and transcriptome in cholangiocarcinoma and illustrate the alteration of tumor on different biological levels. Meanwhile, we also identified the clonal structure of each included tumor sample and discovered different clonal evolution patterns related to patients’ survival. Furthermore, we extracted subnetworks that were greatly influenced by tumor clonal/subclonal mutations or transcriptome change. The topology relationship between genes affected by genomic/transcriptomic changes in biological interaction networks revealed that alteration of genome and transcriptome was highly correlated, and somatic mutations located on important genes might affect the expression of numerous genes in close range.

## Introduction

Cholangiocarcinoma (CCA), a heterogeneous malignant tumor currently acknowledged as the second most common primary liver cancer, showed increasing incidents worldwide during past decades. Although CCA is considered as a rare cancer in most countries due to its relative low incidents (lower than 6 cases per 100,000 people), the situations are different in several countries including China and Thailand, where CCA incident reaches an exceptionally high level. Among all CCA cases, intrahepatic cholangiocarcinoma takes up only 10%, while a minority (15%) of these patients were diagnosed with resectable disease status (Cardinale et al., 2018; Rizvi et al., 2018). While the most promising therapeutic strategy for CCA is surgical operation combined with chemo-/radio- therapy, this approach was considered only suitable for early stage CCA and later stage CCA patients often face the difficulty of lacking effective treatment options. Thus, most CCA patients usually suffered from poor prognosis (5-year survival rate less than 10%). Meanwhile, the heterogeneity of tumor on multiple levels (e.g., genomic, transcriptional) often resulted in resistance to therapy, which further intensifies the challenge of CCA treatments. Thus, a thorough evaluation of the landscape on CCA genome and transcriptome could provide clinically related insights into the genesis and progression of CCA.

Just like other tumors, CCA is developed on the basis of acquiring tumor somatic mutations and clonal evolution. When tumor arises and progresses, the acquisition of somatic mutations randomly happened, resulting in different groups of tumor cells with distinct genetic features. The tumor clone, built up with the complicated constitution of groups of tumor cells (which could be referred as subclones), evolves during its development, dynamically changing its structure to better fit the micro-environment (Greaves and Maley, 2012; McGranahan and Swanton, 2017). During this entirely evolutionary process, certain somatic mutations could give tumor cells survival advantage and subpopulation carrying these genomic alterations expanded, while subclones with mutations reducing survival capacity diminished. Thus, deciphering the clonal evolution in CCA could provide valuable information regarding crucial genetic events in tumorigenesis and progression and how different biological pathways might be affected by these genetic events, which in turn could help further understand the intrinsic mechanisms of tumor progression. Indeed, such efforts have been made in other types of cancer including leukemia (Ferrando and López-Otín, 2017) and solid tumors such as hepatocellular carcinoma (Chen et al., 2018) and breast cancer (Hoadley et al., 2016), and different clonal evolution patterns have been discovered with high correlation with patients’ clinical course.

However, the evolutionary process in CCA still requires further investigation. What more, although the importance of clonal evolution is widely acknowledged, how tumor clonal structure affects tumor transcriptome remained poorly explored. Understanding how somatic mutation interacted with such transcriptome change could further provide valuable insights into the evolutionary mechanism of CCA development. To explore the genetic and transcriptional landscape of intrahepatic CCA, we performed whole exome sequencing and transcriptome sequencing on tumor and corresponding peritumor tissue of 9 CCA patients. The differences on genetic and transcriptional levels were investigated and tumor clonal evolution was deciphered to discover the molecular pathways taking part in the deregulation of tumor cells. These findings will be of great value in understanding the mechanism of CCA development and how transcriptome interact with genetic alterations.

## Materials and Methods

### Sample Collection

Tumor and corresponding peritumor tissue samples were collected from 9 patients diagnosed with intrahepatic cholangiocarcinoma during their surgical operation for tumor removal. The detailed clinical information is provided in Table 1. All human tissue sample collection procedures and usage of these samples were approved by the Institution Review Board of Mengchao Hepatobiliary Hospital of Fujian Medical University and written consents were obtained from all participated patients included in this study.

**TABLE 1:** Clinical characteristics of 9 enrolled CCA patients.

Clinicopathological variables	Patient number (*n* = 9)	Percentage (100%)
**Sex**		
Male	5	55.6
Female	4	44.4
**Age at first enrolled year**, Mean ± SD	62.44 ± 11.78	
**HBV infection**		
Negative	6	66.7
Positive	3	33.3
**HBV DNA**		
≤10^3^	7	77.8
10^3^–10^4^	1	11.1
10^4^–10^5^	1	11.1
**Maximal tumor size**, cm		
0–2.5	2	22.2
2.5–5.0	2	22.2
5.0–10	5	55.6
**Tumor number**		
Single	8	88.9
Multiple	1	11.1
**Liver cirrhosis**		
Absent	5	55.6
Present	4	44.4
**Microvascular invasion**		
Yes	3	33.3
No	6	66.7
**PVTT**		
Yes	1	11.1
No	8	88.9
**Microsatellite lesion**		
Absent	8	88.9
Present	1	11.1
**TNM**		
I	5	55.6
II	1	11.1
IV	3	33.3
**BCLC**		
0	1	11.1
A	4	44.5
B	1	11.1
C	3	33.3

*PVTT, Portal vein tumor thrombosis; TNM, The TNM Classification of Malignant Tumors; BCLC, the Barcelona Clinic Liver Cancer staging system.*

### Whole Exome/Transcriptome Sequencing

Whole-exome and transcriptome sequencing were performed to capture the genetic and transcriptional features for the acquired tumor and corresponding peritumor tissue on Illumina HiSeq 3000 system.

### Whole Exome Sequencing Data Processing

Somatic single nucleotide variants (SNV) and copy number alterations (CNA) were detected for the whole exome sequencing data of tumor tissue samples using the corresponding peritumor as control. To identify SNVs, SomaticSniper (version 1.0.5.0) (Larson et al., 2012) were applied using default parameters provided in the algorithm manual and only SNVs with somatic score ≥ 40 were accepted for downstream analysis. The identified SNVs were further filtered with such criteria to rule out possible false discovery: (1) read depth ≥ 50 in both tumor and peritumor tissues; (2) variant allele frequency ≥ 10% in tumor tissue; (3) variant allele frequency < 10% in normal peritumor tissues. The detected SNVs were then annotated using wANNOVAR to obtain related gene and functional information. For CNVs, TitanCNA (version 1.17.1) (Ha et al., 2014) was applied on the tumor tissue’s whole exome sequencing data using the corresponding peritumor as control using the workflow script provided by the algorithm.

### Transcriptome Sequencing Data Processing

All acquired Transcriptome sequencing reads were first aligned to ribosomal rRNA sequences to remove ribosomal RNA sequence. The unmapped reads were then aligned to human genome reference (GRCH37) using star with GENCODE gene annotation. The gene expression was quantified with fragments per kilobase of exon per million mapped fragments (FPKM) and genes with no read counts in > 50% samples were not included in downstream analysis. Differentially expressed genes were identified using limma package. Genes with adjusted *p* value < 0.05 (Benjamini-Hochberg correction) and fold-change >2 or <0.5 were then considered as significantly differentially expressed between CCA tumor and peritumor.

### Clonal Evolution in CCA

For each CCA tumor sample, inference of subclonal population was conducted using Sclust (Cun et al., 2018). Sclust provided a copy-number analysis method incorporated with mutational clustering to accurately determines copy-number states and subclonal populations. In brief, whole exome sequencing data of the paired tumor and peritumor samples were first processed using command bam process to extract the read ratio and SNP information. Then, the copy number analysis is conducted with command cn for each patient, using the obtained read ratio and SNP information together with SomaticSniper mutation calling results. Finally, the mutational clustering was performed using command cluster based on above results to identify tumor clonal structure.

### Discovery of Altered Subnetworks Influenced by Somatic Mutations and Transcriptome Change

HotNet2 was applied to discover altered subnetworks in the large gene interaction networks. HotNet2 required two input files for subnetwork identification: Heat scores and Interaction network. For somatic mutations, Heat scores for HotNet2 were generated based on mutation distribution across all patients; For transcriptome, Heat scores were generated based on the adjusted *p*-value produced by DESeq2 package. Network hint + hi2012 and irefindex9 provided by HotNet2 was used as the Interaction network for this analysis. The algorithm was run using all recommended parameters provided by algorithm authors and the identified subnetworks were visualized using Cytoscape (version 3.4.0) (Shannon et al., 2003).

## Results

### Case Summary

**FIGURE 1:**
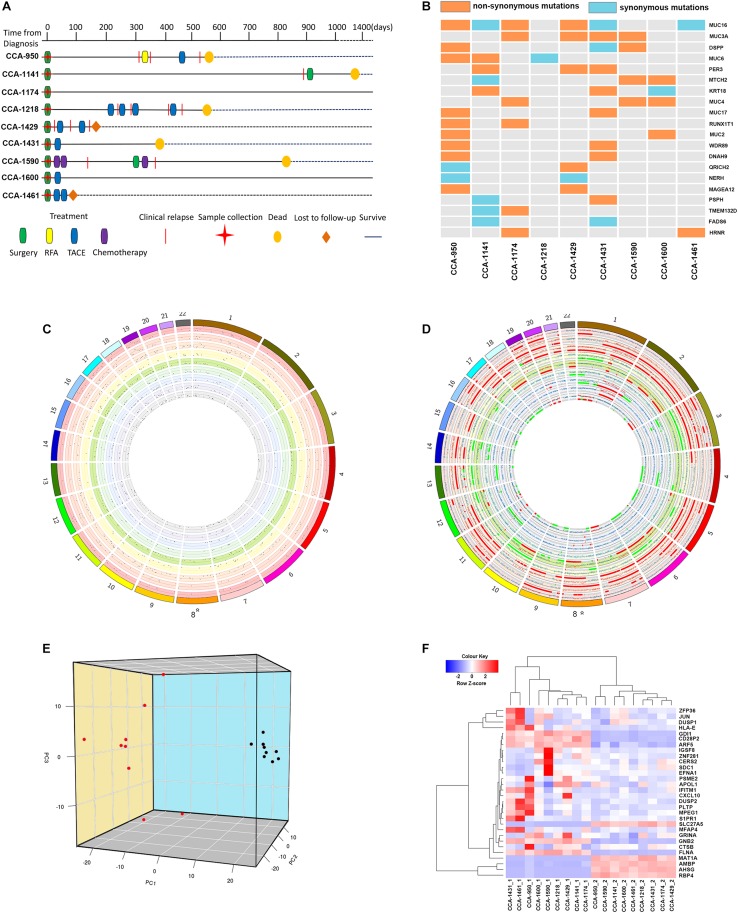
The Clinical courses and the genome and transcriptome landscape of CCA. **(A)** The clinical course of 9 included CCA patients. RFA, Radiofrequency ablation; TACE, Transarterial chemoembolization. **(B)** The common mutated genes with somatic SNVs identified in include CCA patients. Different color indicated the functional type of somatic SNVs in these genes (orange: non-synonymous mutation; light blue: synonymous mutation; gray: not mutated). **(C)** The genomic distribution of somatic SNVs for included CCA patients. Each circle represented a single patient. Dots in the dot plot represented identified somatic SNVs and their heights indicated corresponding variant allele frequencies. **(D)** The genomic distribution of somatic CNVs for included CCA patients. Each circle represented a single patient. The scatter plot showed the logR value for each segment, and regions with different color indicated their copy number status (red: copy number gain; gray normal; green: copy number loss). **(E)** Principal component analysis of CCA transcriptome. The image showed the three-dimension distribution of each sample on the first three principal components. Red dots represented peritumor samples and black dots represented tumor samples. **(F)** Clustering of included tissue samples using top genes correlated with the first three principal components. Genes names and sample names were provided.

In total, 9 patients that were diagnosed with CCA and received surgical operation in Mengchao Hepatobiliary Hospital were included in this study. According to previous reports regarding inflammatory context of liver tumors (Bishayee, 2014; Banales et al., 2016), we chose peritumor tissue as sequencing control to better capture the CCA characteristics. During their surgery, cholangiocarcinoma tumor tissues along with corresponding peritumor tissues were collected and the tumor existence for all patients was histologically confirmed. Then, whole-exome and transcriptome sequencing were performed for acquired tissue samples. Among all included patients, 77.8% (7/9) were diagnosed with TNM staging I-II and the other 22.2% were diagnosed with TNM staging III. The average diameter of tumor in each patient was 5.1 cm (range, 2.0–9.5 cm), while Vascular tumor thrombus was seen in 44.4% (4/9) of all patients. Detailed clinical information for all included patients before they received surgical operation is presented in Table 1 and the corresponding clinical courses were demonstrated in Figure 1A.

### Landscape of CCA Genome and Transcriptome

Whole-exome sequencing achieved a mean average depth of 194.67 × cross all collected tissue samples. To identify tumor somatic mutations, SomaticSniper was applied on all tumor tissue samples using corresponding peritumor as control. Meanwhile, copy number variation was identify using TitanCNA. In total, an average of 378 somatic SNVs (range, 260–529) were detected in tumor tissues, and the distribution of SNVs across human Genome was visualized in Figure 1C. Annotation of acquired SNVs revealed a number of common mutated genes across tumor samples, containing several known cancer-related genes (Figure 1B). Several members of mucin (MUC16, MUC3A, MUC6, and MUC4) were among the most frequently mutated genes, which is consistence with previous reports (Chang et al., 2006; Pereira et al., 2016; Liu et al., 2018; Pareja et al., 2019). Other noteworthy genes included DSPP, PER3, MTCH2, and KRT18, all have been reported with important roles in tumor formation and development. On the other hand, a number of copy number of variations was also identified in tumor samples, showing a wide-spread instability of cancer genome (Figure 1D).

Meanwhile, transcriptome sequencing revealed a significant change on transcriptional level, with a total of 2366 differentially expressed genes identified between CCA tumor and peritumor samples. To provide a clear classification based on samples’ transcriptional features, principal component analysis was conducted to better characterize these samples. Not surprisingly, tumor samples and peritumor samples were well divided by the first three principal components, which explained 21.96%, 10.60%, and 8.68% of variation in samples’ transcriptome, respectively (Figure 1E).

The results showed that the top genes positively associated with PC1 included RBP4, SLC27A5, and PCK2, all of which were known tumor-related genes and correlated with cancer patients’ survival (Anderson and Stahl, 2013; Leithner et al., 2014, 2015; Balsa-Martinez and Puigserver, 2015). Meanwhile, PC1 negatively associated genes included FLNA, ARF5, and SLC25A6, suggesting its connection to cancer development (Savoy and Ghosh, 2013; Casalou et al., 2016; Shao et al., 2016; Cho et al., 2019). For PC2, top positively correlated genes included IFITM1 and GPX1, both have been reported to be associated with risk of numerous cancers (Ravn-Haren et al., 2006; Arsova-Sarafinovska et al., 2009; Lee et al., 2012; Ogony et al., 2016), while most negatively PC2 correlated genes included common-known tumor over-expressed genes such as EFNA1 (Nakamura et al., 2005; Xiang-Dan et al., 2010).

As in PC3, most noteworthy genes positively correlated with this principal component are ZFP36 and DUSP1, both are known for their function of regulation in cancer progression (Montorsi et al., 2016; Nagahashi et al., 2018). Other important correlated genes included t CXCL9 and CXCL10, and they served as important regulators of immune activation in tumor microenvironment (Bronger et al., 2016; Ding et al., 2016; Tokunaga et al., 2018).

Using top genes correlated with the first three principal components, transcriptome clustering revealed that tumor sample and peritumor samples could be indeed well separated (Figure 1F), suggesting that CCA tumors indeed have distinct gene expression patterns compared to peritumor tissues.

### Clonal Evolution in CCA

**FIGURE 2:**
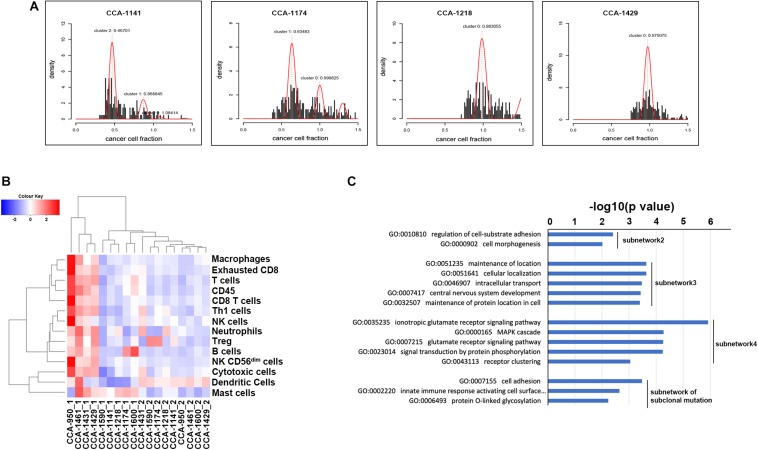
Clonal evolution of CCA and its influence on biological interaction network. **(A)** Mutation clusters identified by Sclust in 4 of 9 included CCA patients. Additional cluster(s) other than cluster 0 were subclonal mutation clusters. Patient identifiers were provided above each plot. **(B)** Clustering of included tissue sample using known immune signatures. **(C)** Go term enrichment results in biological pathways for each identified subnetwork. Subnetwork 2–4 indicated subnetwork altered by clonal mutations and subnetwork of subclonal mutation indicated the subnetwork altered by subclonal mutations. Subnetwork 1 only contained two genes and did not show significant enrichment in Gene Ontology of biological pathways.

To explore the evolutionary process driving tumorigenesis and development, Sclust algorithm was applied to infer subclonal populations in cancer genomes. Combining copy-number analysis and mutation clustering approach, Sclust could accurately determine copy-number states as well as cellular prevalence of mutations. As shown in Figure 2A and Supplementary Figure S1, different types of clonal structure were revealed. For 7 of the included patients (CCA-1218, CCA-1431, CCA-1461, CCA-950, CCA-1429, CCA-1590, and CCA-1600), no subclonal mutations were identified since all mutations within each sample could be clustered into one single cluster according to their allele frequencies. These results showed that during the tumor clonal evolution of these patients, the randomly accumulated mutations might not create subclones with significant survival advantage. The other 2 patients (CCA-1141 and CCA-1174), on the other hand, presented considerable portion of subclonal mutations. In patient CCA-1141, two large subclonal mutation clusters were observed, with cellular frequency of 46.70% and 86.88%, respectively. The other patient, CCA-1174, also showed one considerable subclonal mutation clusters, accounting for 63.48% of all tumor cells. The existence of a large number of subclonal mutations might suggest that the emerge of these tumor subclones took place in the later stage of tumor development, while a high cellular frequency further indicated that they possessed notable survival advantage. Surprisingly, these two patients with subclonal mutations identified showed better prognostic outcome compared to other patients, with relapse-free survival and over-all survival both longer than 20 months. One possible explanation is that in this kind of patients, some critical mutations that might greatly benefit tumors’ growth took place in the later period of tumor development (which explained the expanding tumor subclones), while other tumor acquired these genetic alterations in the early stage, and thus resulted in the differences in patients’ prognosis. Evaluation of known immune signature based on gene expression further revealed that CCA-1141 and CCA-1174 could be categorized into cold tumor with relatively low level of cells correlated with immune response (Figure 2B). This result suggested that the clonal evolution of CCA might be closely related to its immune microenvironment, and high level of infiltration might suppress the evolutionary process of tumor cells.

**FIGURE 3:**
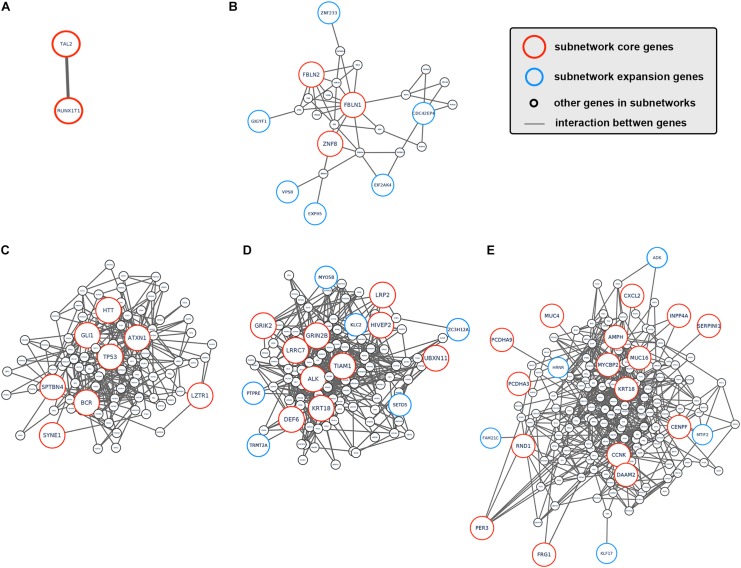
Subnetworks altered by CCA clonal and subclonal mutations. **(A–E)** Subnetworks that were affected by tumor clonal/subclonal mutations identified by HotNet2. Red circles indicated genes that were identified as core genes within corresponding subnetwork, and blue circles indicated expansion genes within corresponding subnetwork, while gray circles indicated genes that were not identified by HotNet2 but served as linker genes that connect identified genes.

To better understand how tumor clonal evolution affected different biological pathways/processes in tumor cells, we first divided patients’ somatic mutations into clonal mutations and subclonal mutations, and then HotNet2 algorithm was used to scan gene interaction networks for altered subnetworks affected by different categories of mutations. For clonal mutations, four subnetworks were identified (Figures 3A–E). The first subnetwork contained only 2 core genes: RUNX1T1 and TAL2 (Figure 3A). These two genes were both related to gene transcription and their dysregulation has been reported to promote tumorigenesis in various cancer. The second subnetwork (Figure 3B) contained three core genes (FBLN1, FBLN2, and ZNF8, label with red) and six expansion genes (CDC42EP4, EIF2AK4, EXPH5, GIGYF1, VPS8, and ZNF233, labeled with blue). Gene Ontology (GO) term enrichment analysis revealed that this subnetwork is closely related with extracellular matrix structure, cell-substrate adhesion and cell morphogenesis (Figure 2C), suggesting that tumor clonal mutation would show a tendency to affect biological pathways related to cells’ interaction with microenvironment, which is critical for tumor development. The third subnetwork was made up of eight highly interacted genes, namely ATXN1, BCR, GLI1, HTT, LZTR1, SPTBN4, SYNE1, and TP53 (Figure 3C). All these genes were known as oncogenes, including a well-known driver gene in various cancer, TP53. The last and biggest subnetwork (Figure 3D) including 10 core genes (ALK, DEF6, GRIK2, GRIN2B, HIVEP2, KRT18, LRP2, LRRC7, TIAM1, UBXN11) and 6 expansion genes (KLC2, MYO5B, PTPRE, SETD5, TRMT2A, and ZC3H12A), most of which served as important components of multiple signaling pathways and involved in regulation of cancer cell.

Interestingly, several subnetworks altered by tumor clonal mutations were closely related to major metabolism pathways. It’s within expectation since one well-known intrinsic character for tumor cells is its abnormal metabolism.

On the other hand, we also analyzed the subnetwork affected by tumor subclonal mutations. Considered that two out of nine patients were identified with subclonal mutations, HotNet2 identified only one subnetwork that was altered by subclonal mutations (Figure 3E). GO analysis revealed that the mutated genes were most relevant to cell adhesion. This result suggested that subclonal mutations benefiting tumor metastasis might bring survival advantage for corresponding tumor subclones.

### Transcriptome Analysis Revealed Alteration in Pathways Enriched in CCA Clonal Evolutionary Process

**FIGURE 4:**
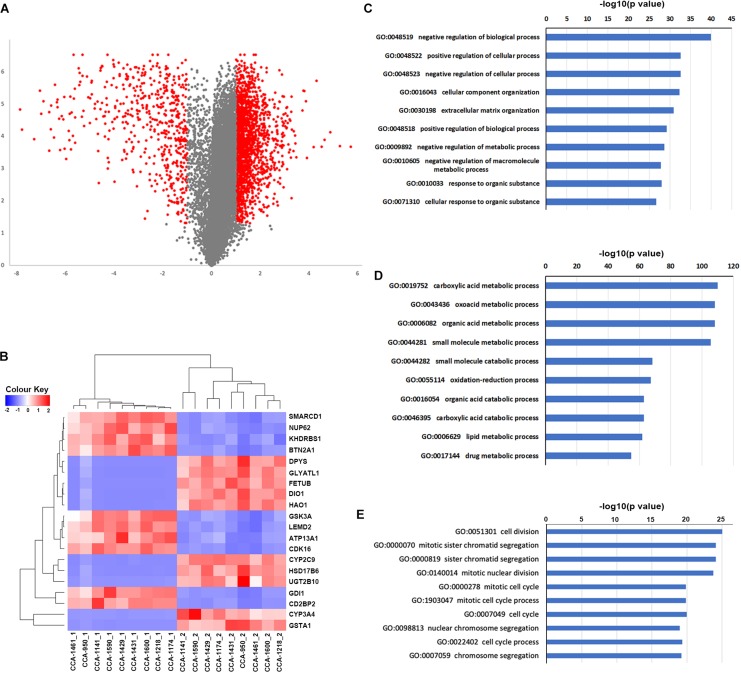
Differentially expressed genes and their connection to tumor clonal evolution. **(A)** Volcano plot showing the differentially expressed genes identified by limma package. Genes with | log2(fold-change) | ≥ 1 and P_*adjusted*_ ≤ 0.05 were marker as red, and other genes were marked as gray. **(B)** Clustering of included tissue samples using top differentially expressed genes. **(C,D)** Go term enrichment results for up-regulated **(C)** and down-regulated **(D)** genes. **(E)** Go term enrichment results in biological pathways for genes in identified subnetworks altered by transcriptome change.

we next explored the transcriptome landscape to evaluate the change in gene expression during CCA development. Using limma algorithm, a total of 2366 differentially expressed genes [| log(fold-change)| ≥ 1 and P_*adjusted*_ < 0.05] were identified in CCA tumor comparing to peritumor samples (Figure 4A). Among these genes, 1833 were significantly upregulated in CCA and 533 were downregulated. Transcriptome clustering using the top 20 differentially expressed genes also showed an excellent separation between tumor and peritumor samples (Figure 4B). GO-term enrichment analysis revealed that the up-regulated genes (Figure 4C) were mostly enriched in the regulation of biological process (GO:0048519, GO:0048522 and GO:0048523), while down-regulated genes (Figure 4D) were mostly enriched in metabolism related biological processes including carboxylic acid metabolic process (GO:0019752) and oxoacid metabolic process (GO:0043436).

**FIGURE 5:**
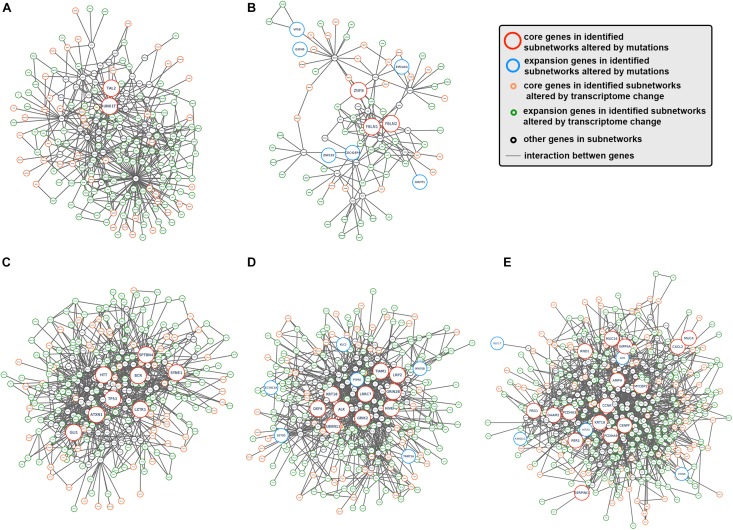
The interaction between somatic mutations and transcriptome change. **(A–E)** Interaction networks formed by subnetworks that were altered by tumor mutation and corresponding close-range subnetwork genes affected by transcriptome change. Red circles indicated genes that were identified as core genes within corresponding subnetwork, blue circles indicated expansion genes within corresponding subnetwork, orange circle indicated core genes in subnetworks altered by transcriptome change, and green circles indicated expansion genes in subnetworks altered by transcriptome change, while gray. circles indicated genes that were not identified by HotNet2 but served as linker genes that connect identified genes.

Next, HotNet2 was once again applied to identify the altered subnetworks affected by transcriptome aberration. Surprisingly, genes identified in subnetworks affected by somatic mutations (clonal or subclonal) rarely appeared in subnetworks affected by transcriptome change. However, mapping genes affected by transcriptome change back to biological interaction networks revealed that many of these genes were in close range of the altered subnetworks affected by tumor somatic mutations (Figures 5A–E). It appeared that tumor genomic alterations created a spreading aberration across the biological interaction network and thus a number of genes were under their influence, resulting in a wide-range change of tumor transcriptome. Meanwhile, Gene Ontology enrichment analysis revealed that subnetworks altered by transcriptome change were dominantly enriched in biological processes related to cell division and cell cycle (Figure 4E), including cell division (GO:0051301), cell cycle (GO:0004857), protein localization (GO:0008104) and cellular component organization (GO:0016043), indicating notable change of proliferation capacity happened during tumor clonal evolution. It’s not surprising that cell morphogenesis (GO:0000902), cellular localization (GO:0051641), intracellular transport (GO:0046907) and maintenance of protein location in cell (GO:0032507), four biological pathways that had been reported to be significantly enriched for mutation-affected subnetworks, were also enriched for these transcriptome-change-affected genes.

Furthermore, we also found that these multi-omics-altered subnetworks were significantly overlapped with pathways presented in kegg database (Supplementary Figures S2–S21). Noteworthily, all hot subnetworks were significantly overlapped with pathways in cancer (hsa05200), while other enriched pathways included cell-cycle (Vermeulen et al., 2003), ECM-receptor interaction (Lu et al., 2012) and VEGF signaling pathway (Roskoski, 2007), all have been reported to be related with tumor progression.

To further investigate if the altered pathways could be clinically related, we obtain the gene expression profile from TCGA-CHOL dataset and use Cox regression analysis to identify potential biomarkers for CCA patients’ overall survival. Univariate cox regression analysis revealed that 14 genes within the hot subnetworks showed expression pattern significantly correlated with patients’ overall survival (Supplementary Figure S22), including PTN and EGFR, two major players in tumor progression. Then these genes were utilized to generate the multivariate Cox regression model using stepwise forward selection. The acquired model consisted of 4 genes (PTPRZ1, CFH, RCN2 and VPS4B) and corresponding model parameters were summarized in Supplementary Table S1. The prognostic value was then calculated from the model score as follows:

$$p\,r\,o\,g\,n\,a\,o\,t\,i\,c\,v\,a\,l\,u\,e=\frac{e^{{}^{s\,c\,o\,r\,e}}}{1+e^{{}^{s\,c\,o\,r\,e}}}$$

Applying the 50-percentage cutoff of prognostic value, the TCGA-CHOL dataset could be divided into two risk groups with distinct prognostic patterns (Kaplan-Meier survival analysis, *p* = 0.00015, Supplementary Figure S23).

All these results suggested that the alteration of tumor genome and transcriptome were closely related, and the influence of driver gene mutations might spread to faraway downstream.

## Discussion

Clonal evolution has been proved to be one of the most important concepts in tumor genesis and development. Currently, a lot of researches have been conducted in variable kinds of tumors and revealed different clonal evolution patterns along with cancer development, providing insights into better understanding of their evolutionary mechanism. These valuable knowledges were of great value in prognosis evaluation and treatment selection. In our analysis including 9 cholangiocarcinoma patients, we discovered that a major portion (7/9) of CCA cases did not show visible subclones within the primary tumors, indicating the existence of mature clonal structure after tumorigenesis. Interestingly, the other two CCA patients with considerable subclones demonstrated significantly longer RFS and OS compared to these patients without visible subclones. Above phenomena might suggest that the forming of a stable and lasting clonal structure at early stage might lead to worse clinical outcome for CCA cases. Another intriguing finding is that the expanding subclones in tumor were connected to relatively low immune signatures (as we showed before), showing a close interaction between tumor and its immune microenvironment. Meanwhile, identification of subnetworks affected by CCA clonal/subclonal mutations revealed that clonal mutations’ influence spread across a number of different biological pathways, while subclonal mutations influence mainly focused on pathways that benefiting tumor metastasis. This result indicated that most mutations with survival advantage were acquired during early stage of CCA development and acquisition of mutations on key regulator genes could affect how tumor evolved.

Cancer development involved biological alteration/dysregulation on multiple biological levels, including genomic, epigenomic and transcriptomic. Although a lot of studies have been conducted on every single omics level, discovering a variety of patterns and mechanism for how these alterations contribute to tumorigenesis, one major question still remained largely unanswered: how the alteration on multiple biological levels interact? In our analysis, we identified key subnetworks that were greatly affected by genomic and transcriptomic changes. Interestingly, although genes in subnetworks greatly affected by genomic change rarely overlapped with those under the influence of transcriptome alteration, it appeared that these two groups of genes were in close range within biological interaction networks, suggesting that dysregulation of genome and transcriptome were closely related. One possible explanation might be that genes that were mutated served as sources of disturbance and affected the expression of their neighbor genes. This disturbance could further spread, creating a large-scale change of tumor transcriptome.

## Conclusion

In conclusion, integrating whole exome and transcriptome sequencing technology, our analysis demonstrated the landscape of CCA genome as well as transcriptome and discovered the different clonal evolution patterns in these patients. We also identified biological pathways significantly altered by tumor somatic mutations and transcriptome change and reveal the connection among the alteration on different omics levels, which could bring insight for better understanding the mechanism of CCA development and help future prognosis evaluation.

## Data Availability Statement

The raw sequence data reported in this paper have been deposited in the Genome Sequence Archive (Wang et al., 2017) in BIG Data Center (Big Data Center Members, 2018), Beijing Institute of Genomics (BIG), Chinese Academy of Sciences, under accession numbers HRA000085, which can be accessed at https://bigd.big.ac.cn/gsa-human.

## Ethics Statement

The studies involving human participants were reviewed and approved by the Institution Review Board of Mengchao Hepatobiliary Hospital of Fujian Medical University. The patients/participants provided their written informed consent to participate in this study.

## Author Contributions

GC, ZC, and HZ contributed the conception and design of the study. GC, JZ, and XH performed the bioinformatic analysis. XD, SL, and ZC performed the sample collection and clinical data collection. HZ, GC, F-EL, and XL interpreted the analysis results. GC and HZ wrote the manuscript. ZC, F-EL, and XL wrote the sections of the manuscript. All authors contributed to manuscript revision, read and approved the submitted version.

## Conflict of Interest

The authors declare that the research was conducted in the absence of any commercial or financial relationships that could be construed as a potential conflict of interest.
