# Supplementary material for: Genomic and Transcriptomic Landscape of Tumor Clonal Evolution in Cholangiocarcinoma
Source: Front Genet. 2020 Mar 13;11:195. doi: 10.3389/fgene.2020.00195 (PMC7083074; doi:10.3389/fgene.2020.00195)
Supplement: Supplementary file 1 [file Presentation_1.pdf]

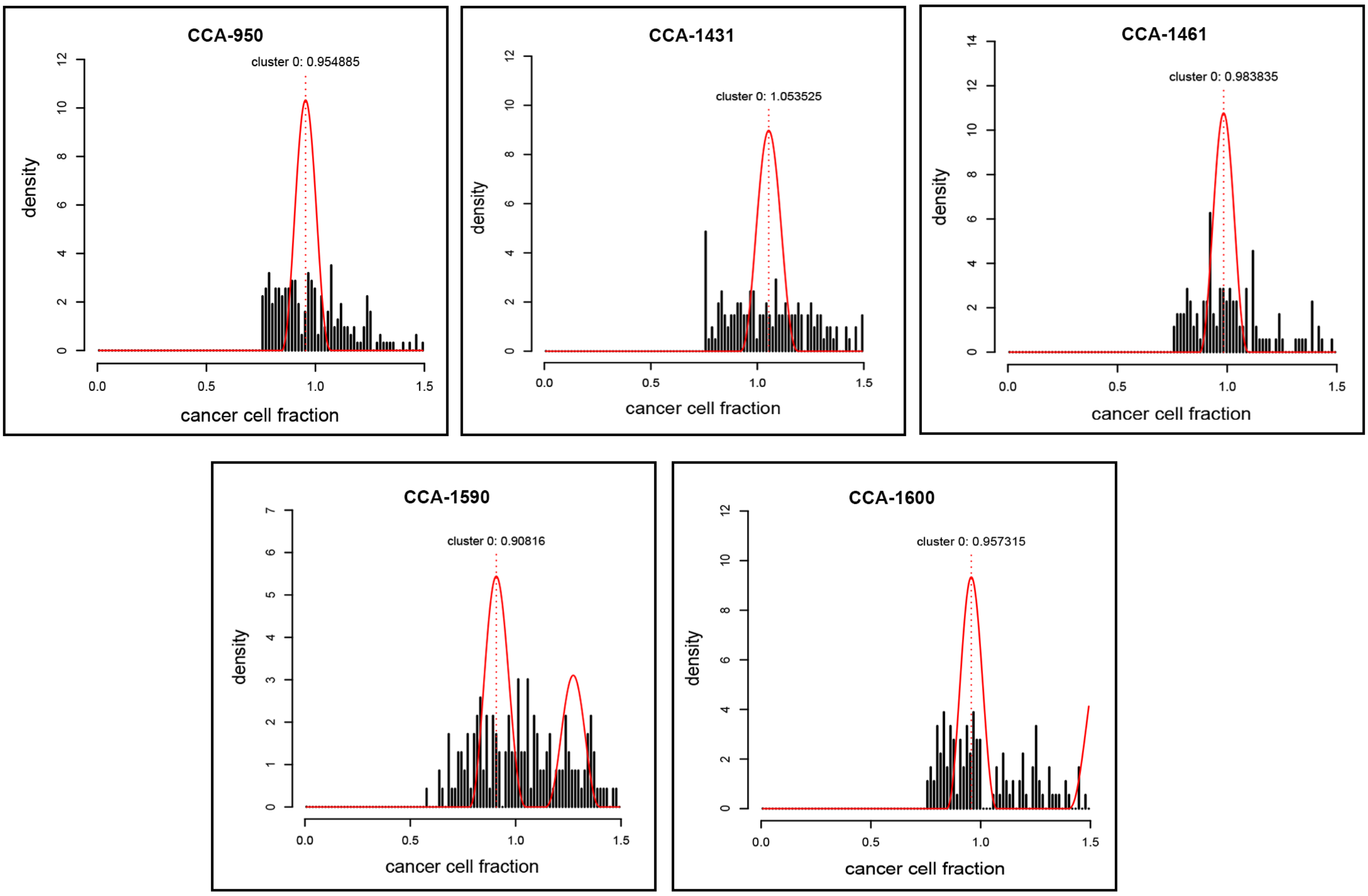

Supplementary Figure S1. Mutation clusters identified by Sclust in 5 of 9 included CCA patients.

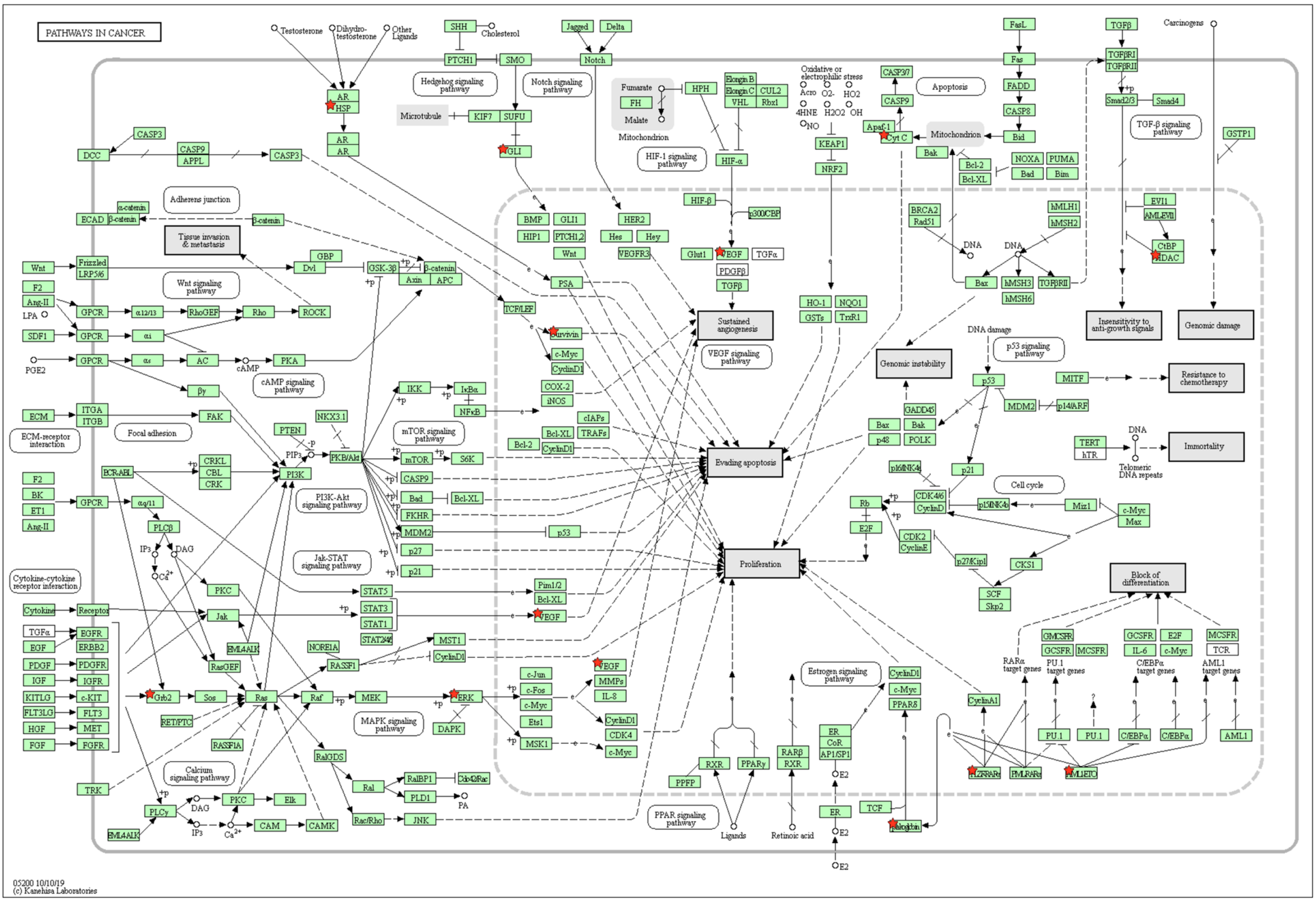

Supplementary Figure S2. Overlap between multi-omics-altered subnetwork 1 and pathway in cancer from KEGG.

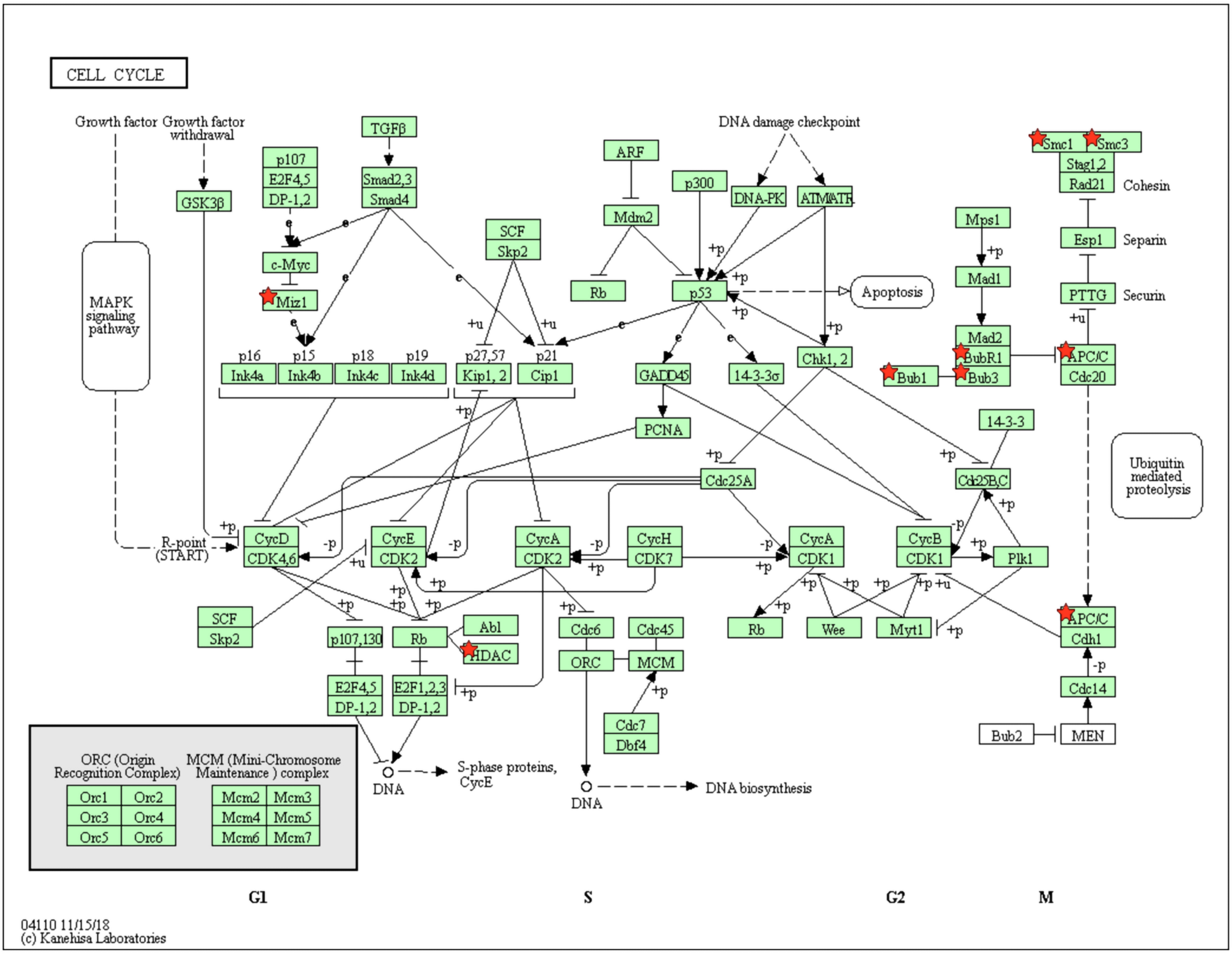

Supplementary Figure S3. Overlap between multi-omics-altered subnetwork 1 and pathway cell cycle from KEGG.

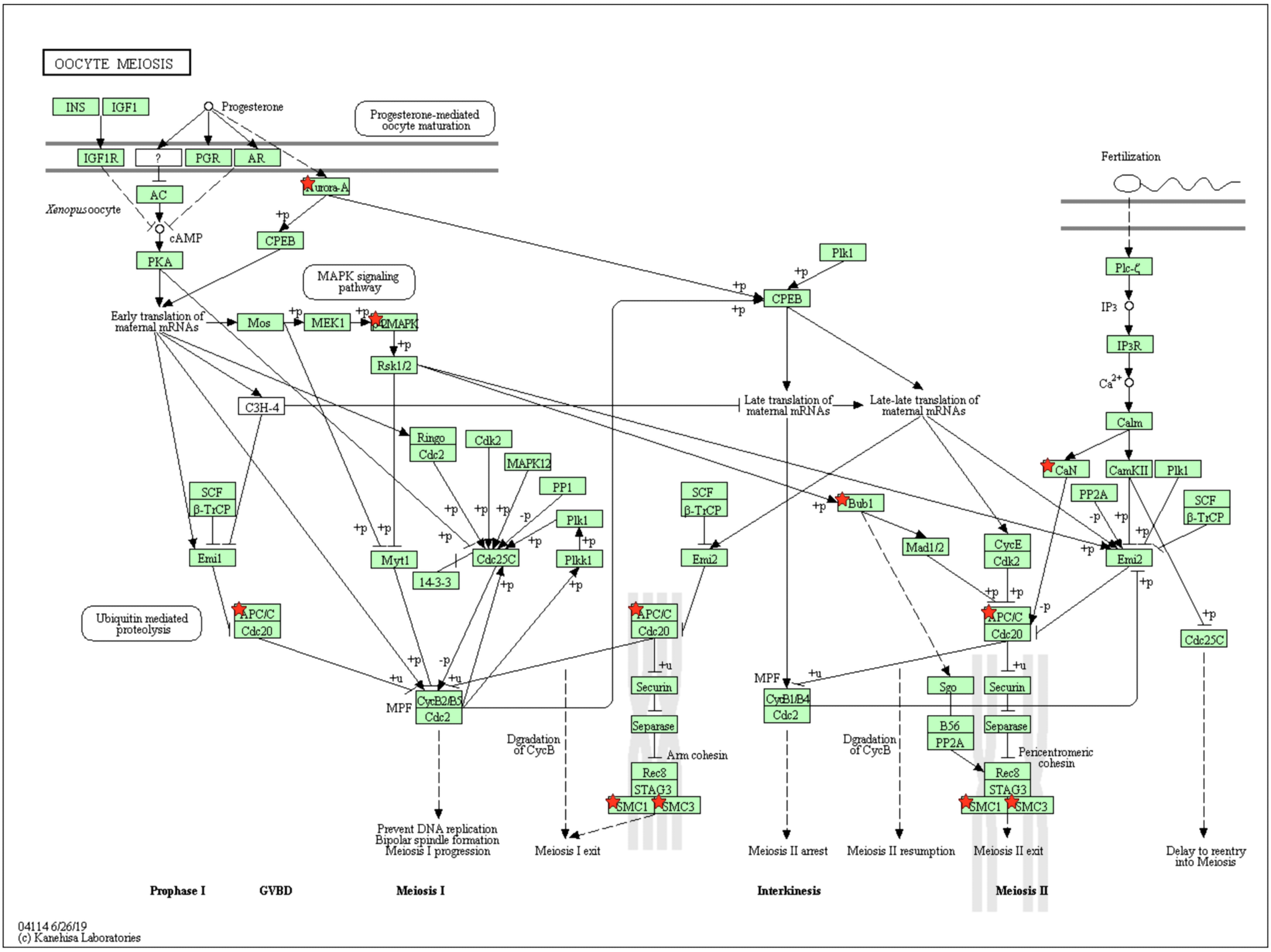

Supplementary Figure S4. Overlap between multi-omics-altered subnetwork 1 and pathway oocyte meiosis from KEGG.

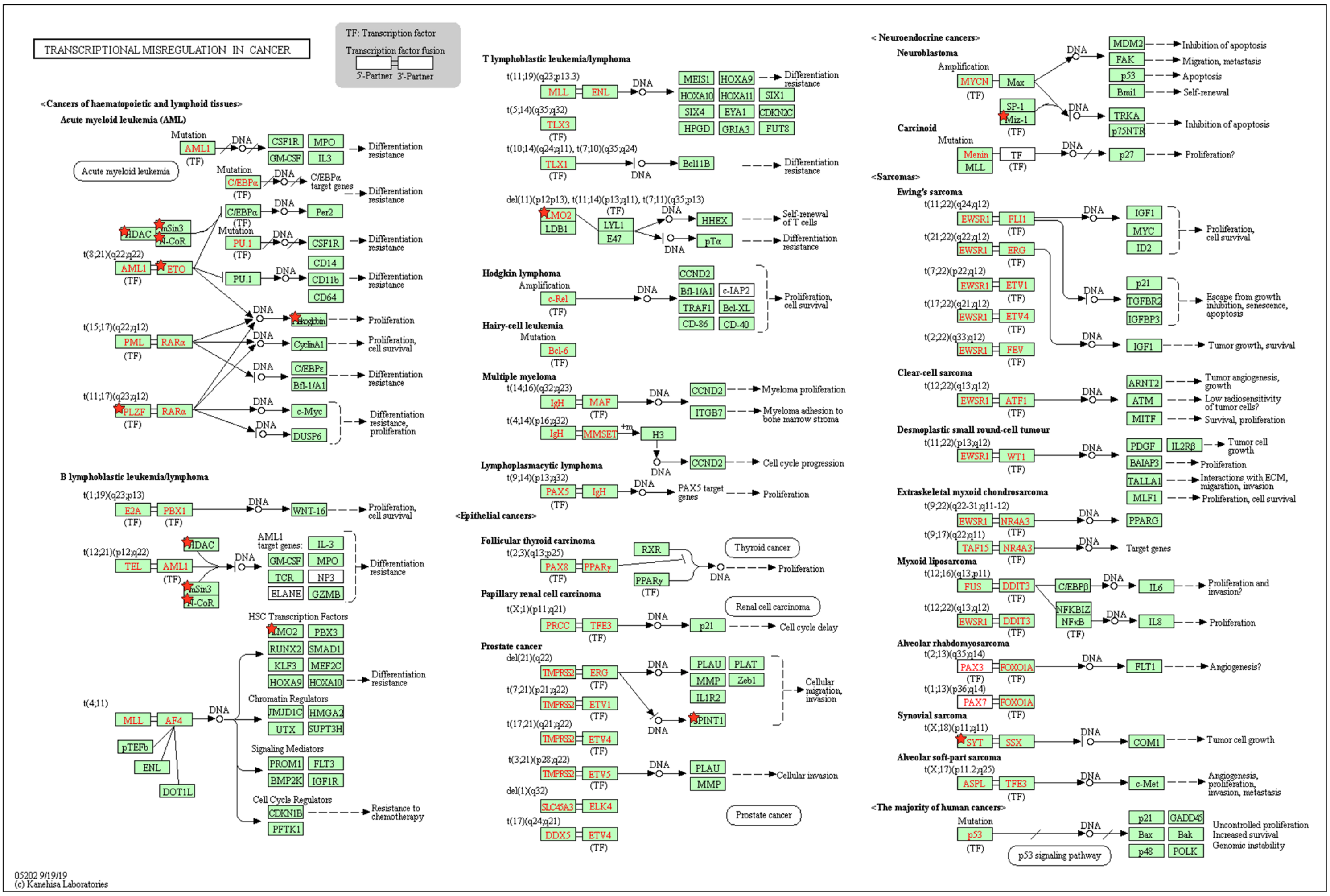

Supplementary Figure S5. Overlap between multi-omics-altered subnetwork 1 and pathway transcriptional misregulation in cancer from KEGG.

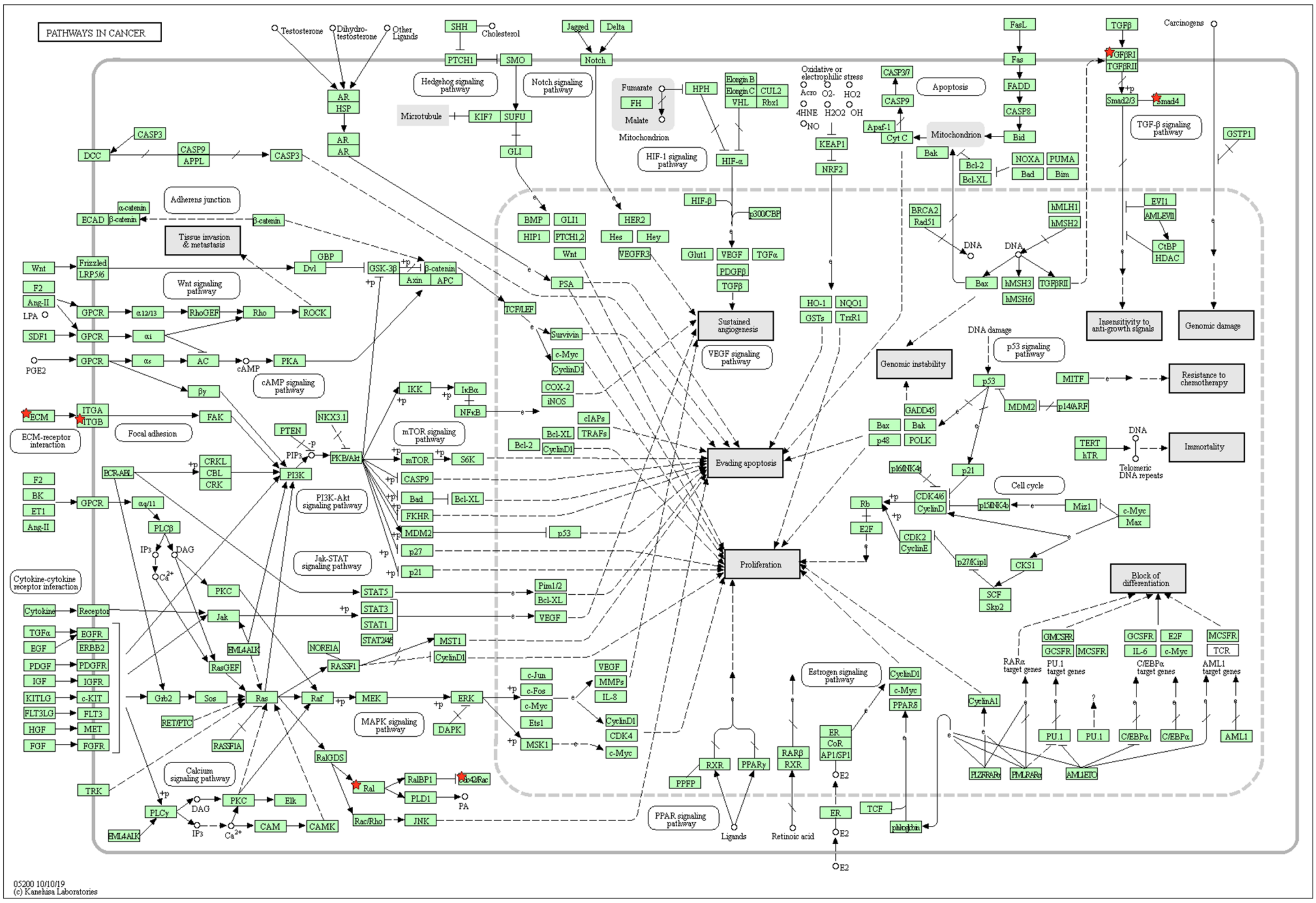

Supplementary Figure S6. Overlap between multi-omics-altered subnetwork 2 and pathway in cancer from KEGG.

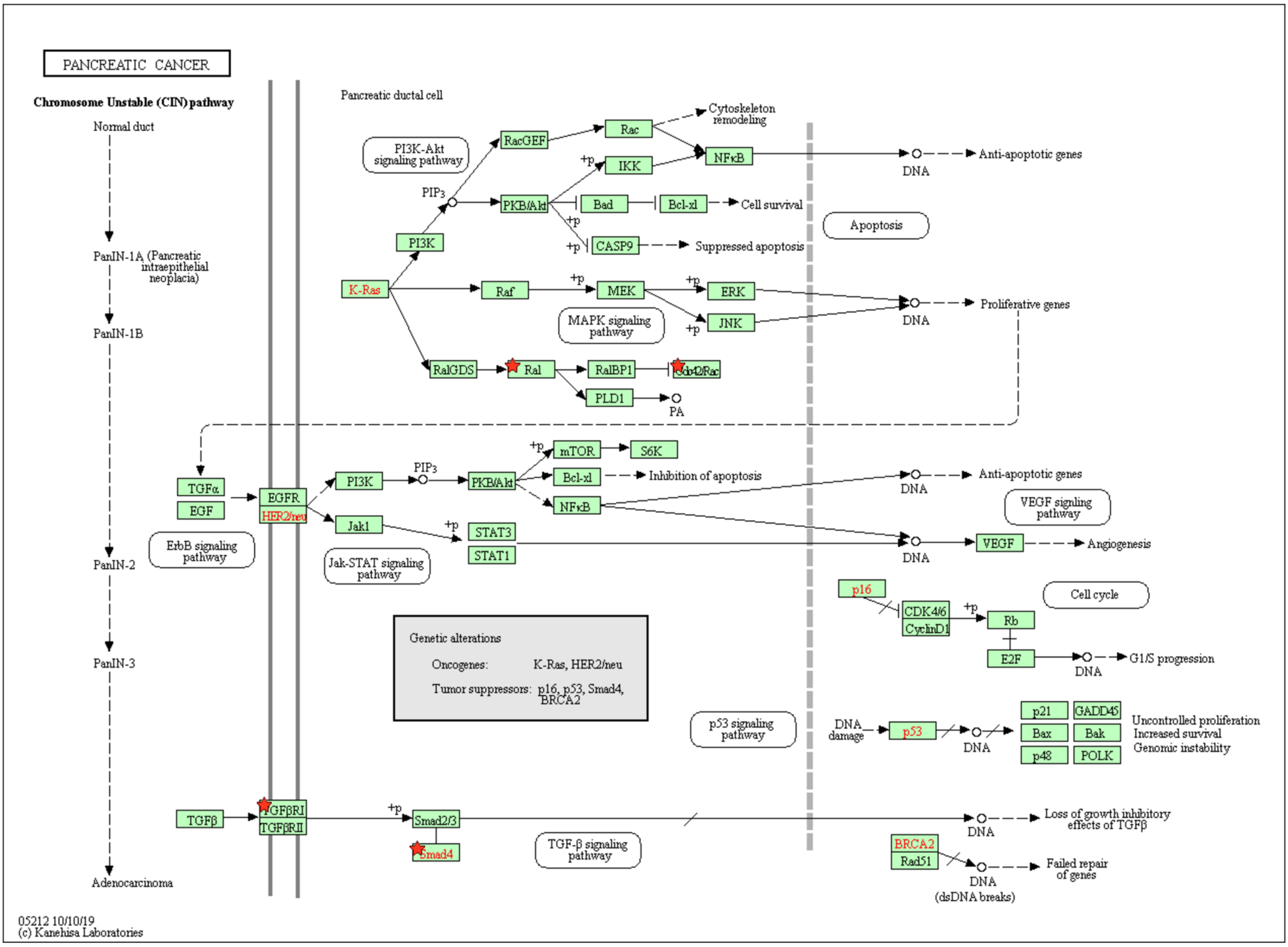

Supplementary Figure S7. Overlap between multi-omics-altered subnetwork 2 and pathway pancreatic cancer from KEGG.

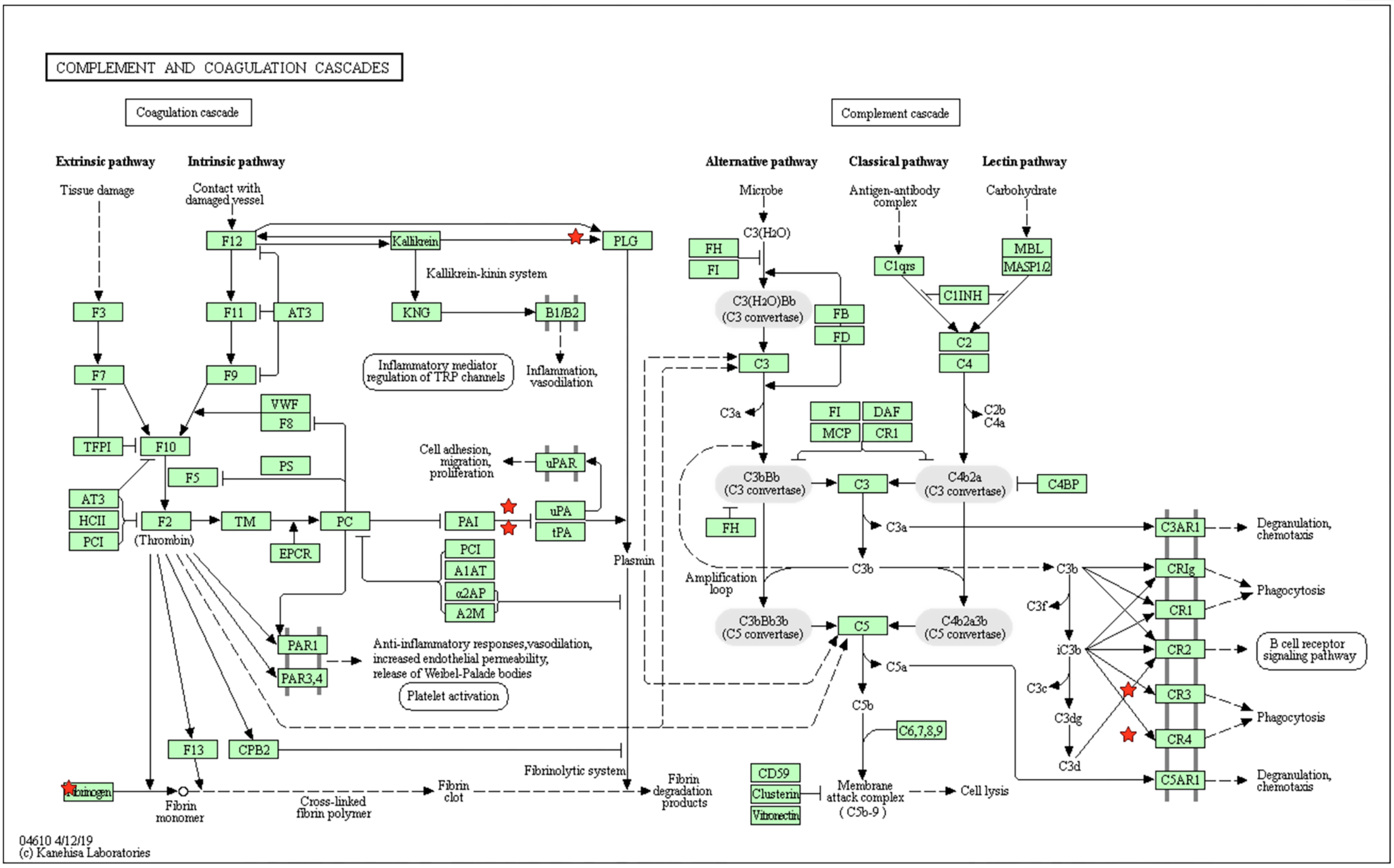

Supplementary Figure S8. Overlap between multi-omics-altered subnetwork 2 and pathway complement and coagulation cascades from KEGG.

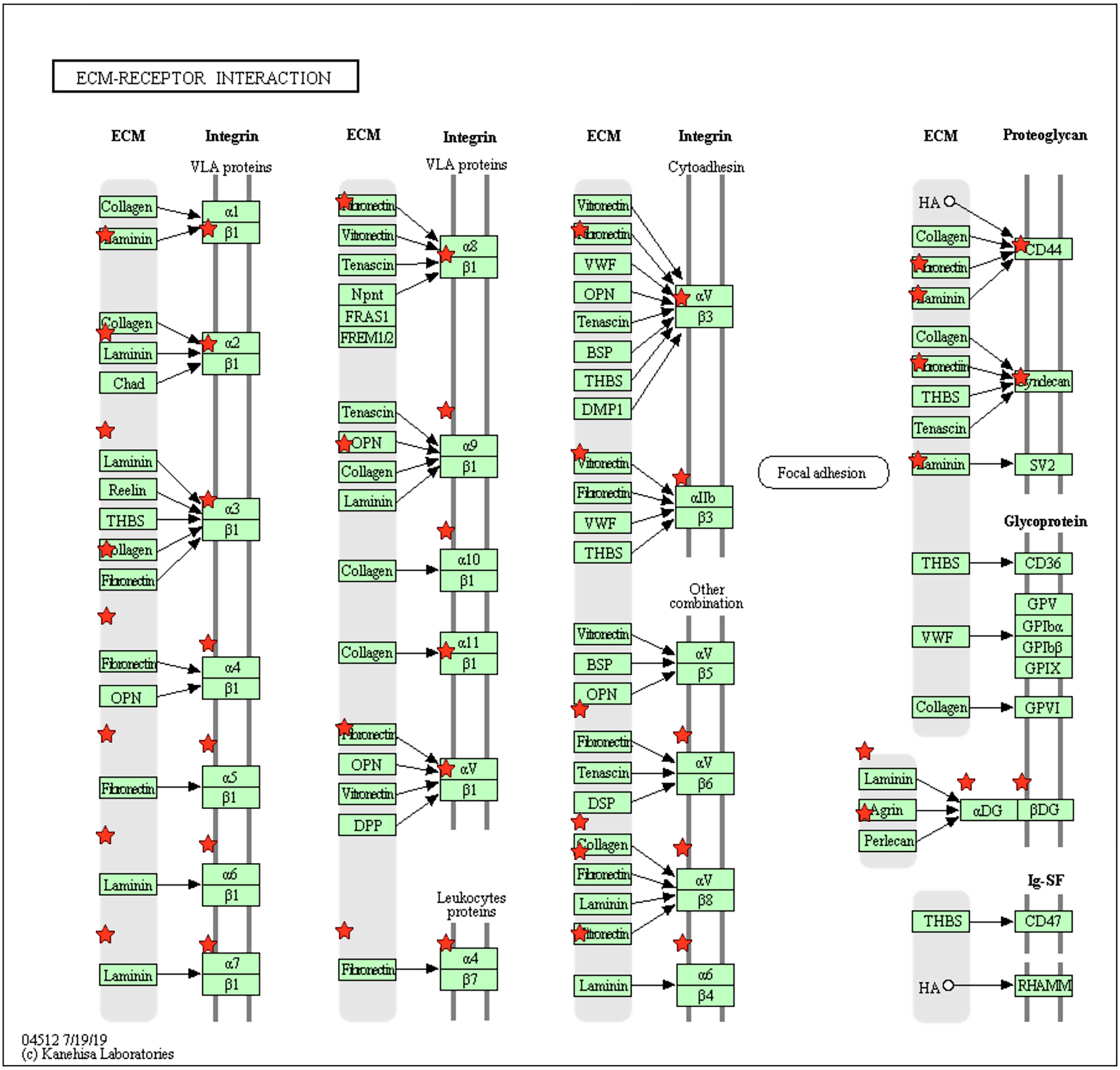

Supplementary Figure S9. Overlap between multi-omics-altered subnetwork 2 and pathway ECM-receptor interaction from KEGG.

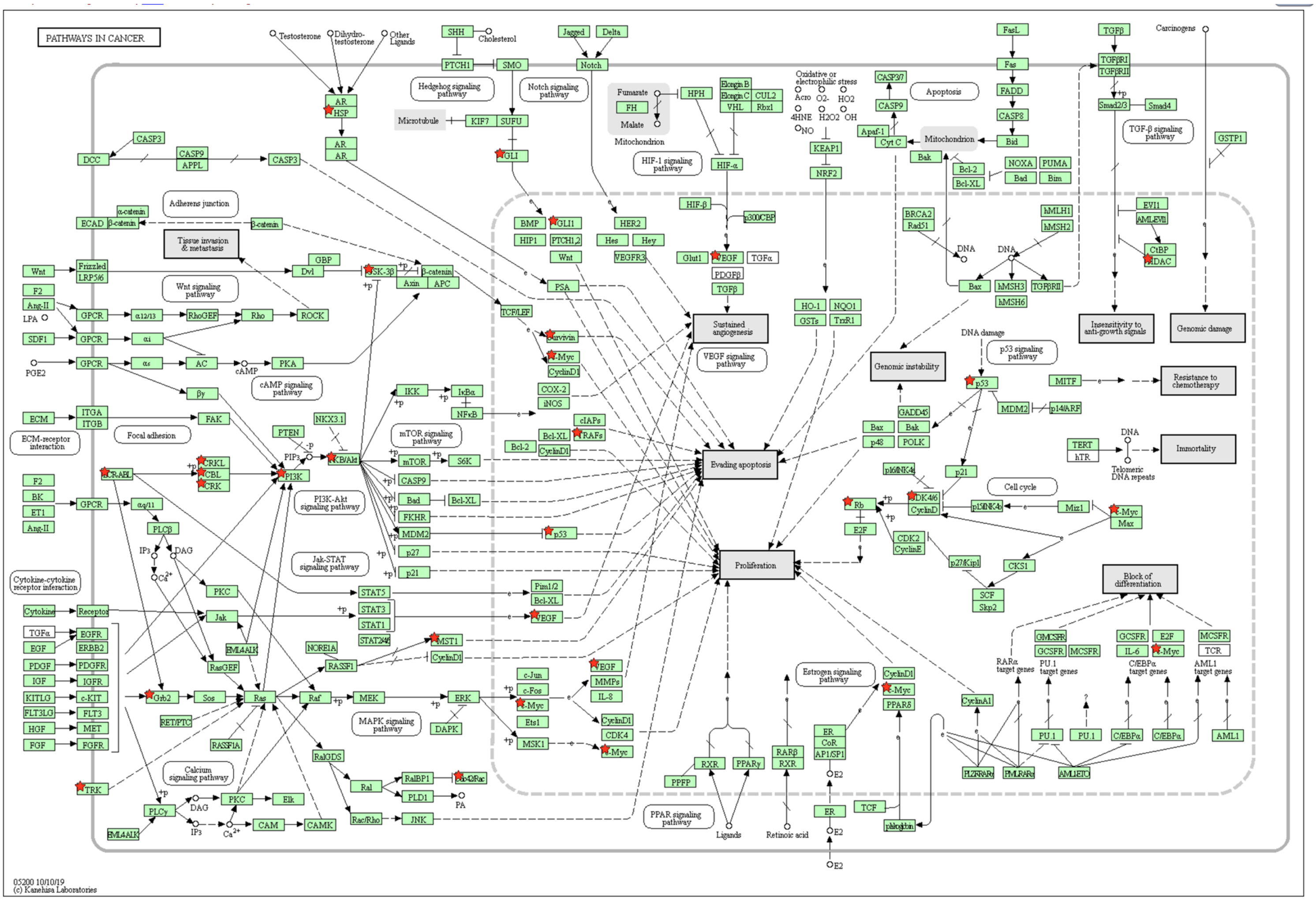

Supplementary Figure S10. Overlap between multi-omics-altered subnetwork 3 and pathway in cancer from KEGG.

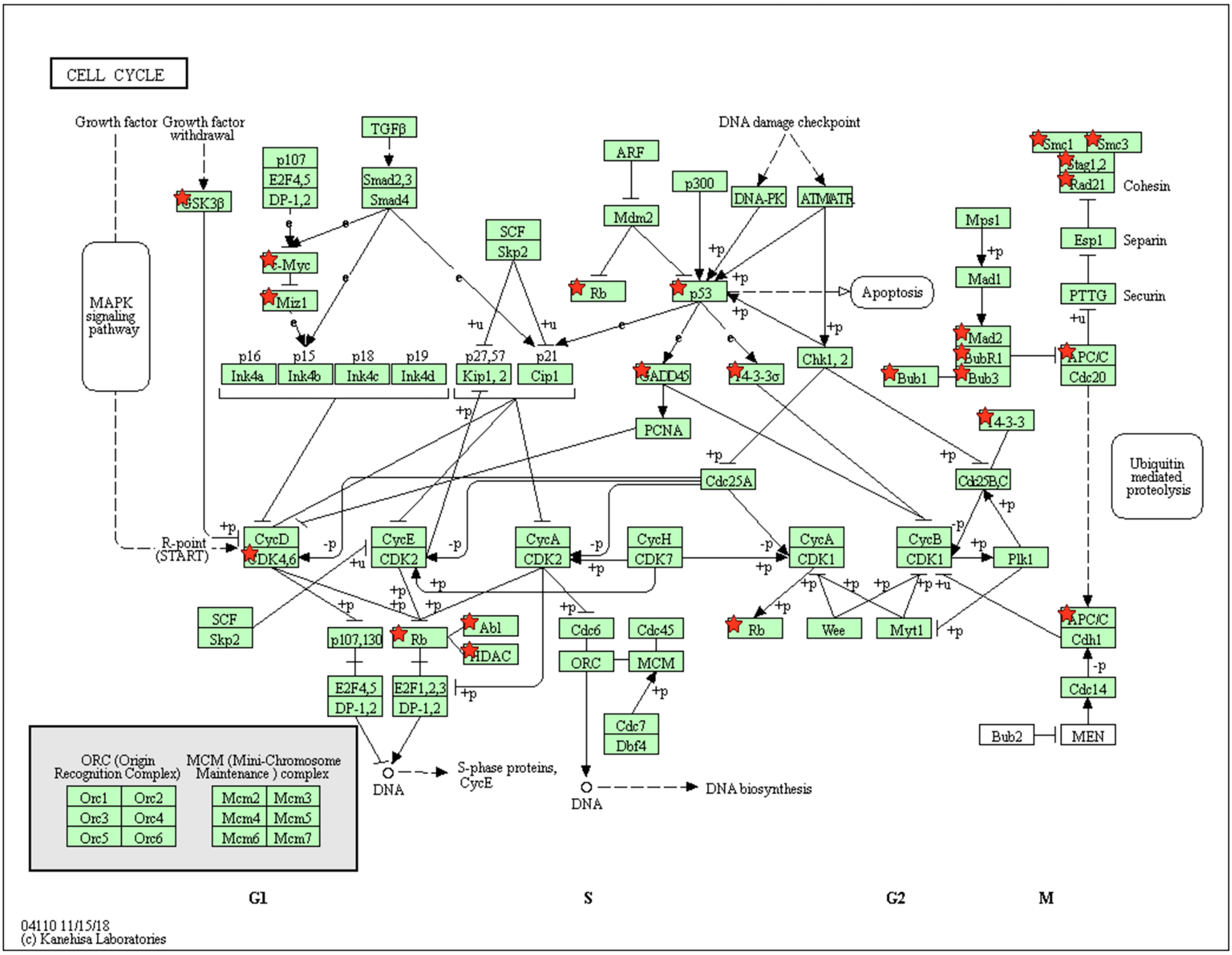

Supplementary Figure S11. Overlap between multi-omics-altered subnetwork 3 and pathway cell cycle from KEGG.

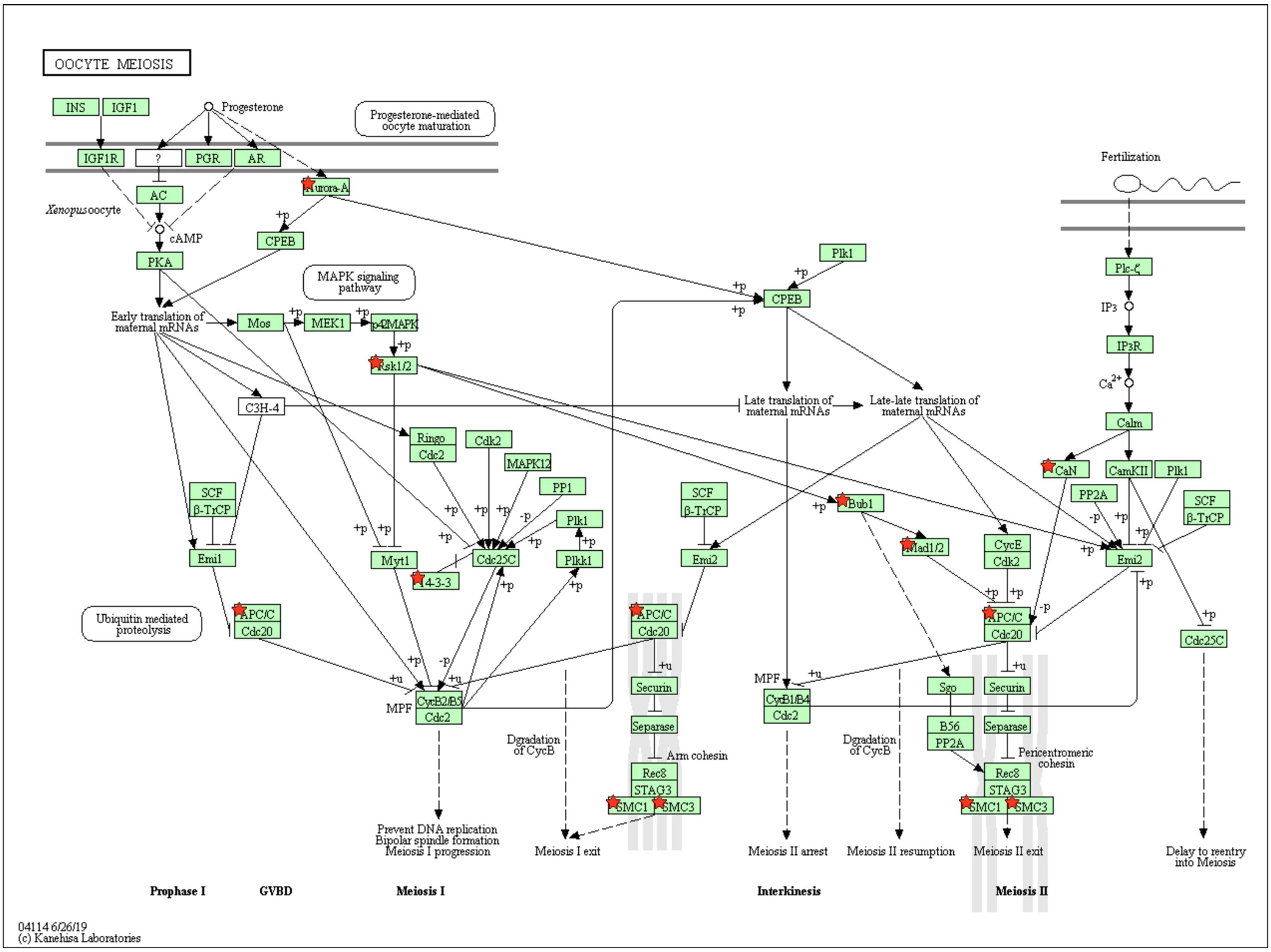

Supplementary Figure S12. Overlap between multi-omics-altered subnetwork 3 and pathway oocyte meiosis subnetwork from KEGG.

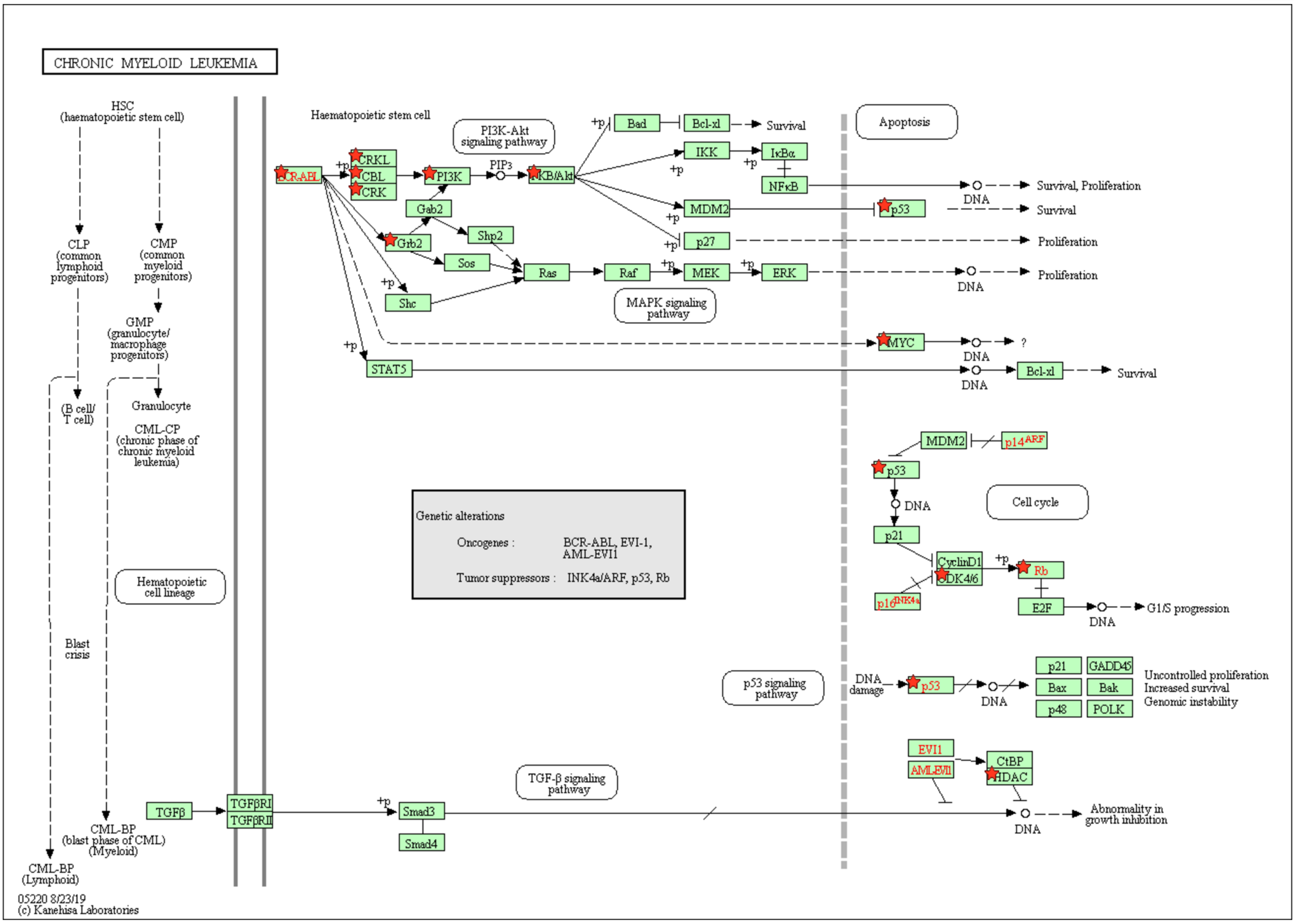

Supplementary Figure S13. Overlap between multi-omics-altered subnetwork 3 and pathway chronic myeloid leukemia from KEGG.

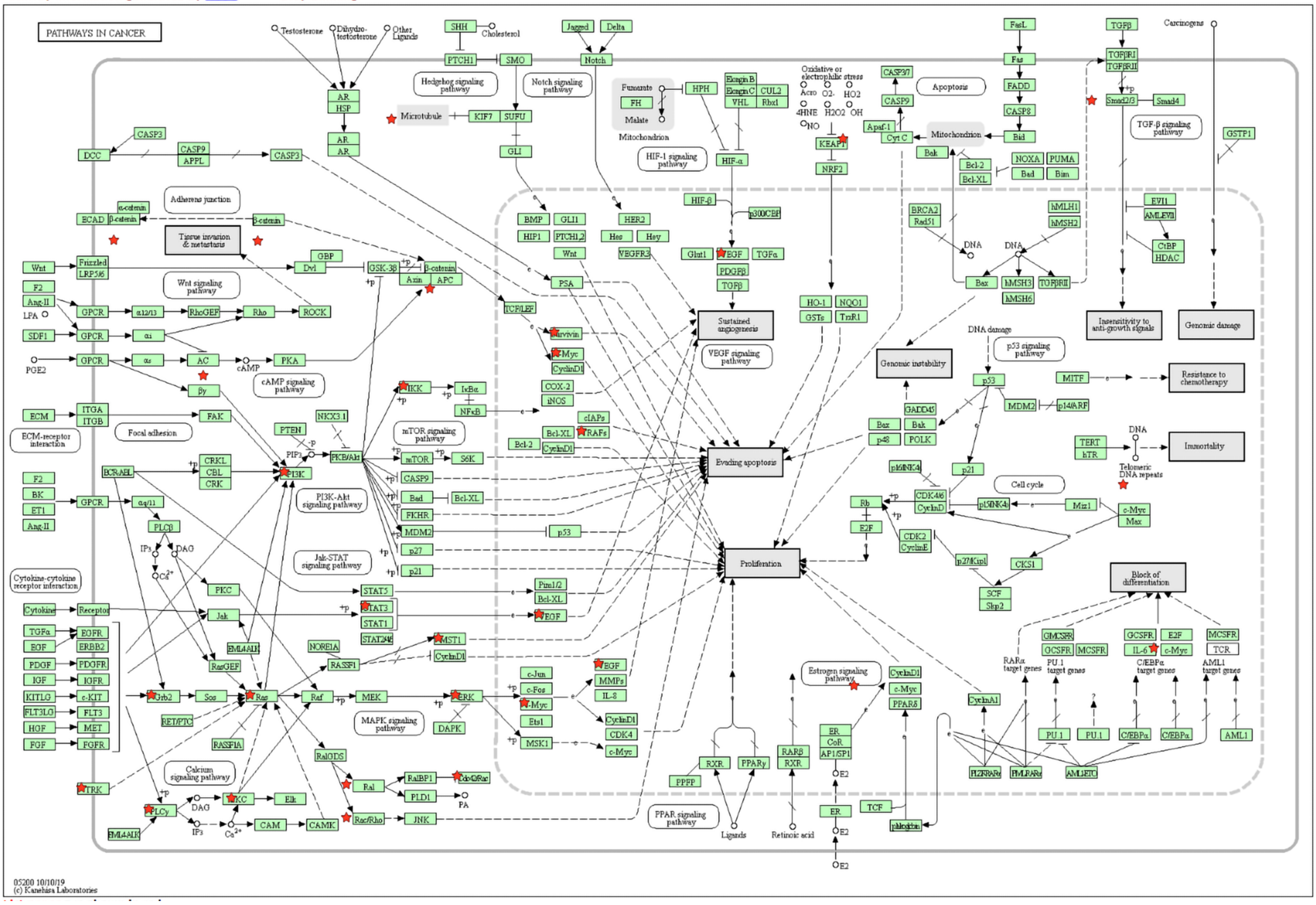

Supplementary Figure S14. Overlap between multi-omics-altered subnetwork 4 and pathway in cancer from KEGG.

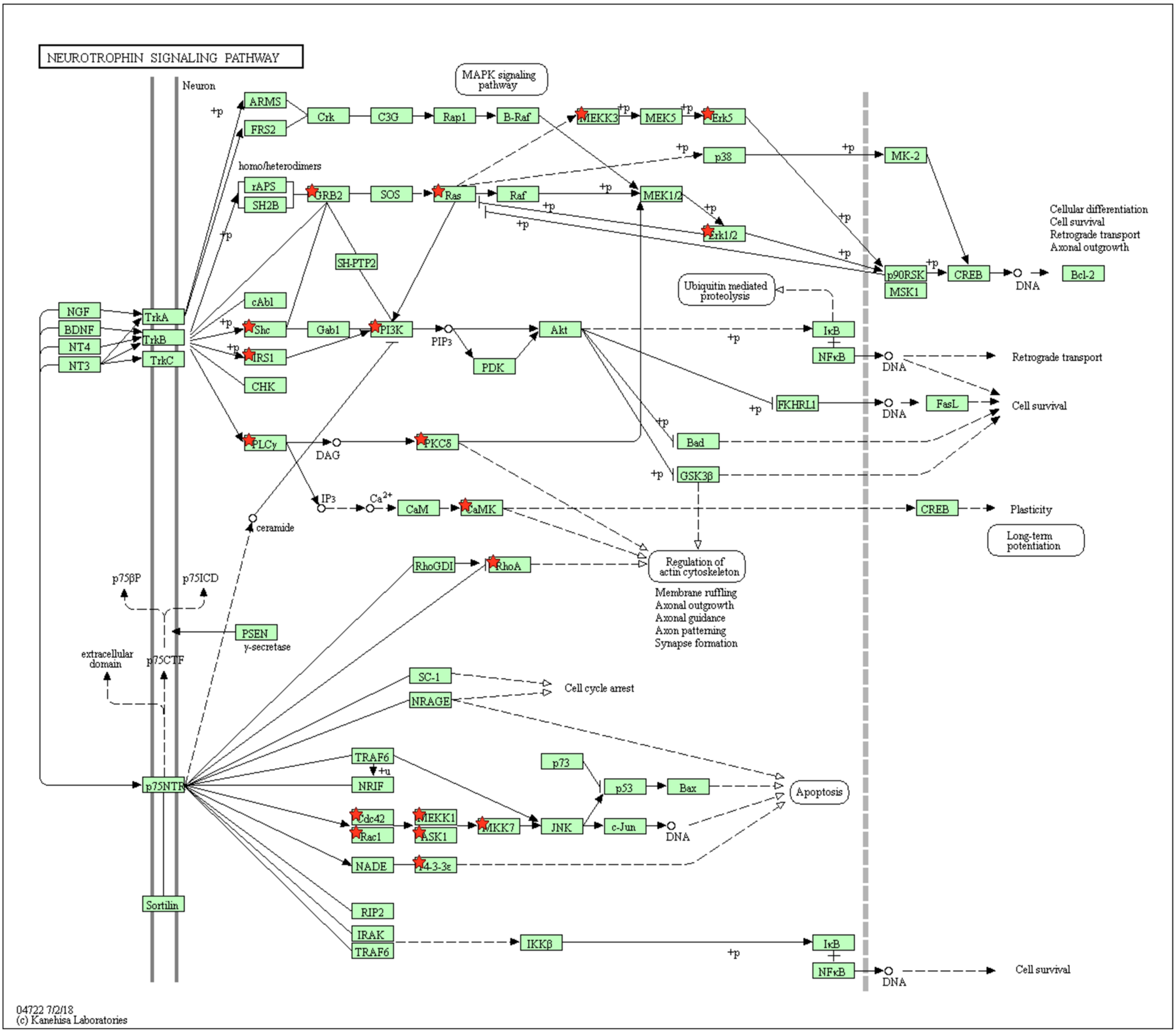

Supplementary Figure S15. Overlap between multi-omics-altered subnetwork 4 and neurotrophin signaling pathway from KEGG.

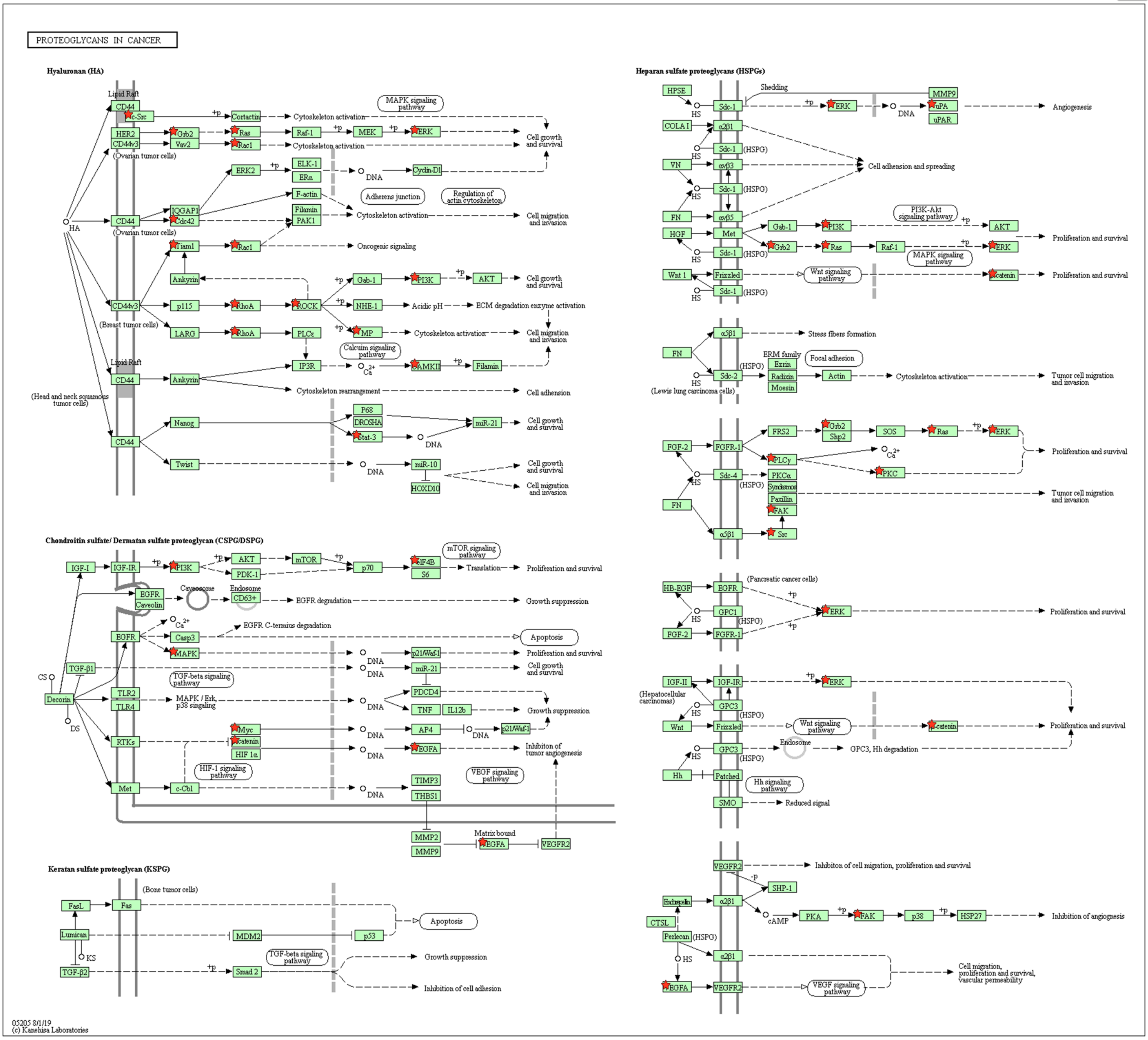

Supplementary Figure S16. Overlap between multi-omics-altered subnetwork 4 and pathway Proteoglycans in cancer from KEGG.

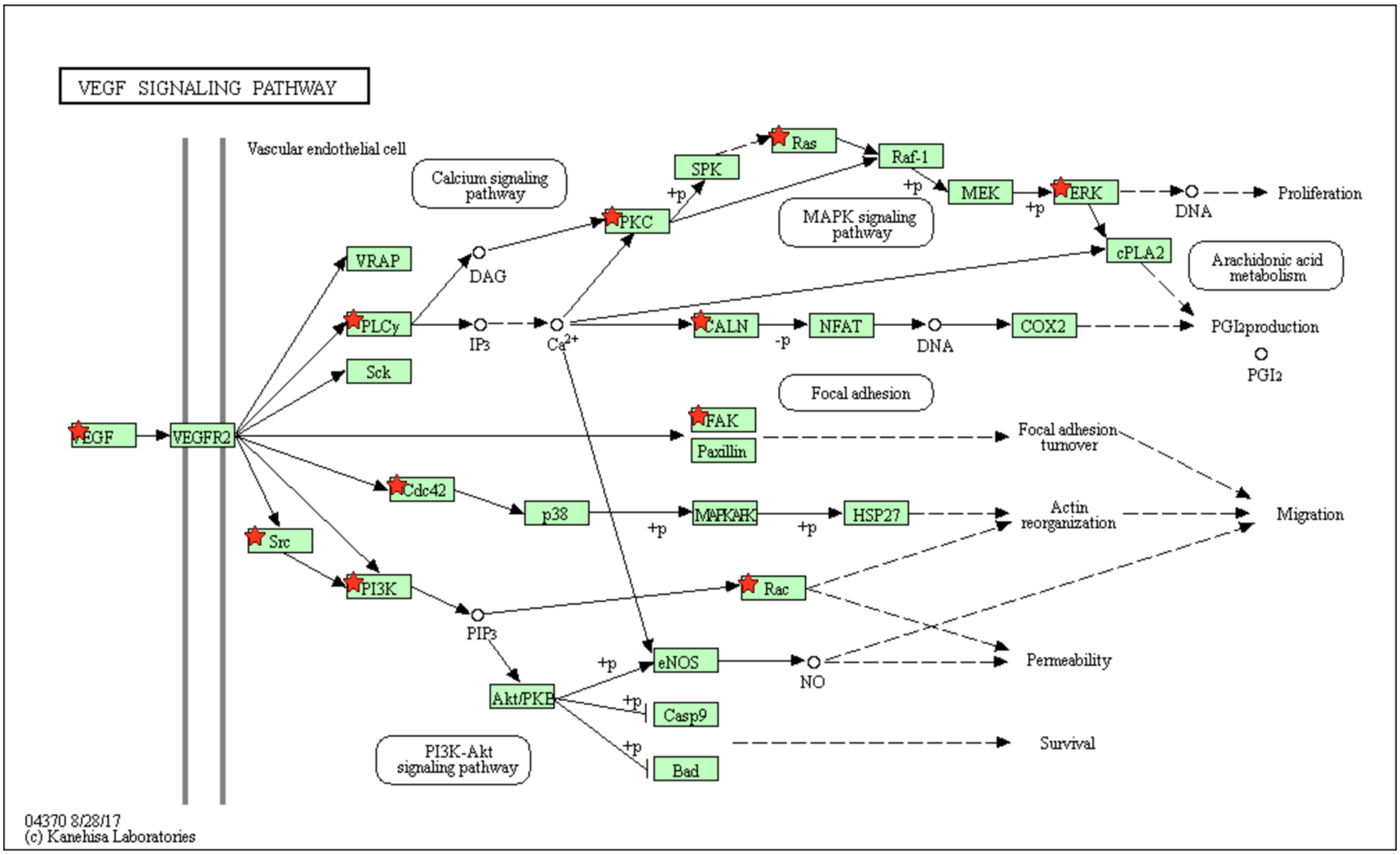

Supplementary Figure S17. Overlap between multi-omics-altered subnetwork 4 and VEGF signaling pathway from KEGG.

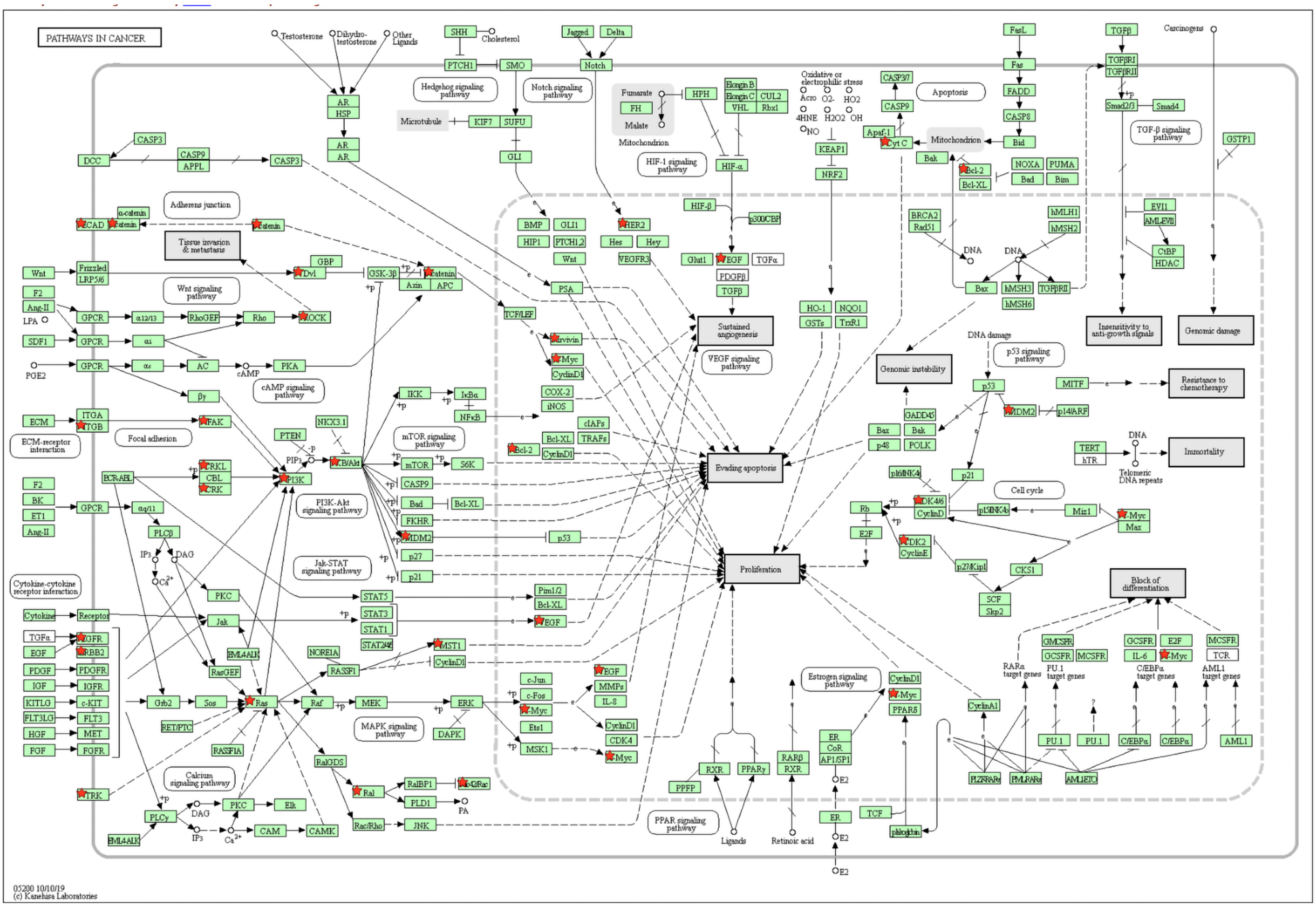

Supplementary Figure S18. Overlap between multi-omics-altered subnetwork 5 and pathway in cancer from KEGG.

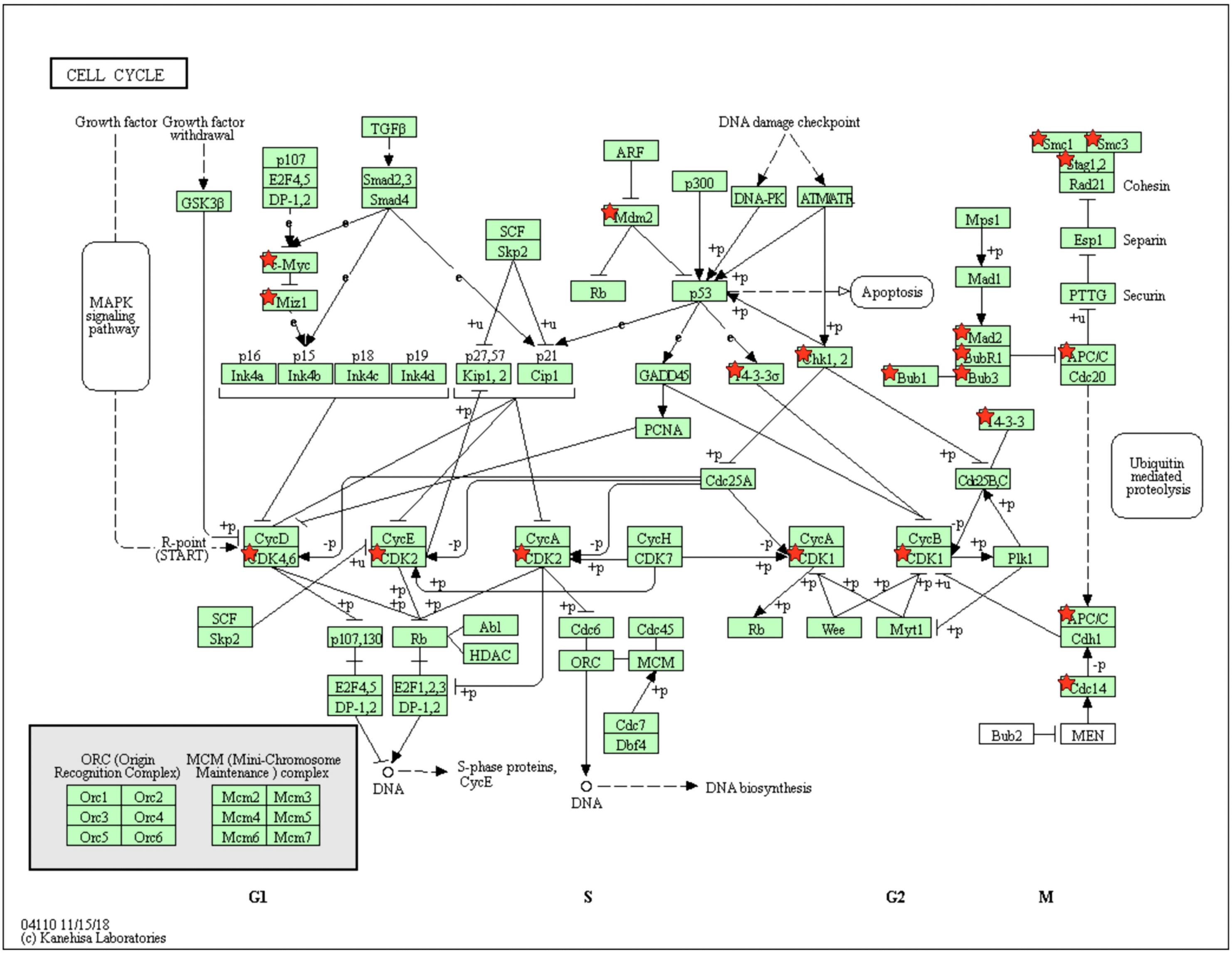

Supplementary Figure S19. Overlap between multi-omics-altered subnetwork 5 and pathway i cell cycle from KEGG.

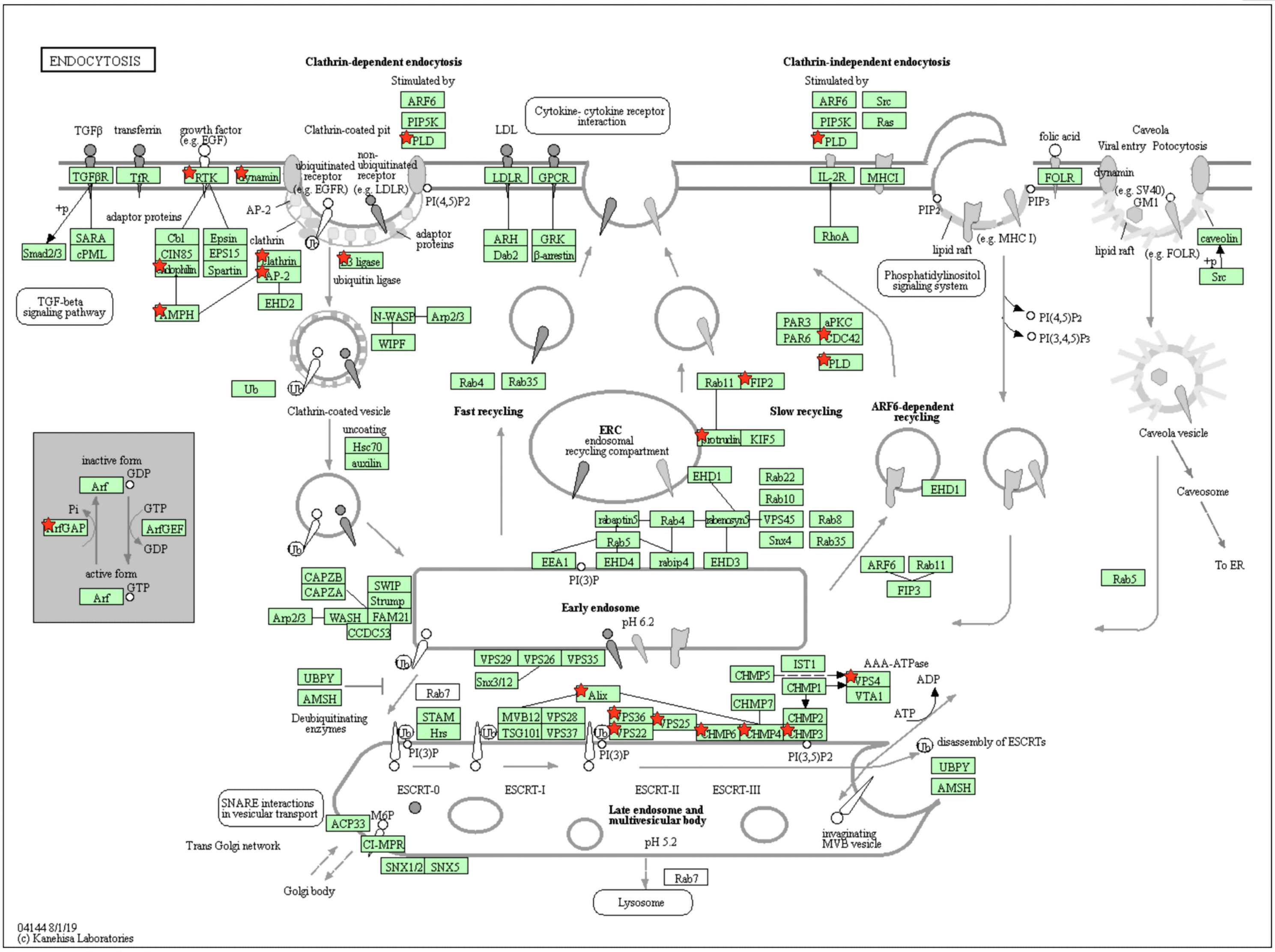

Supplementary Figure S20. Overlap between multi-omics-altered subnetwork 5 and pathway endocytosis from KEGG.

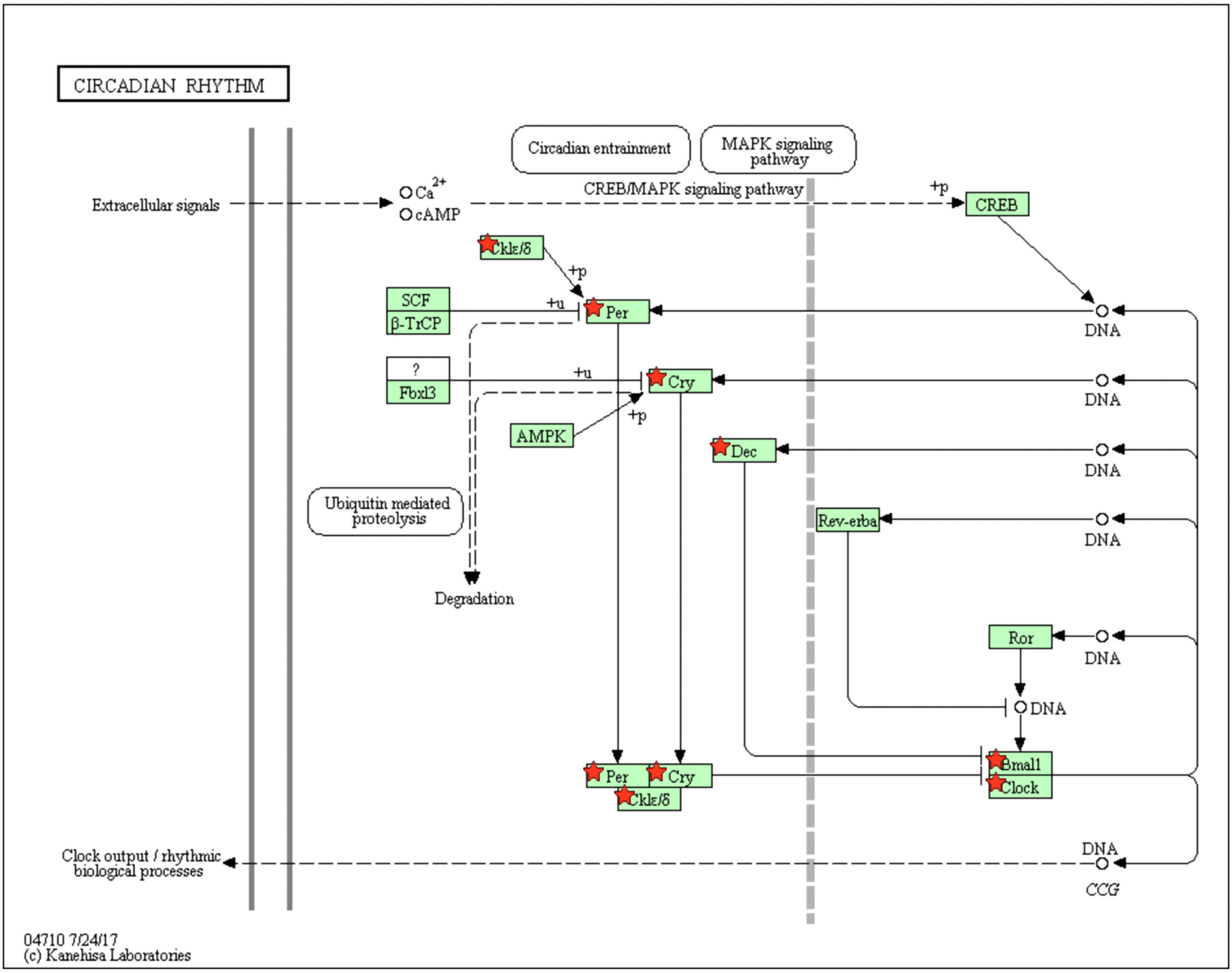

Supplementary Figure S21. Overlap between multi-omics-altered subnetwork 5 and pathway Circadian rhythm from KEGG.

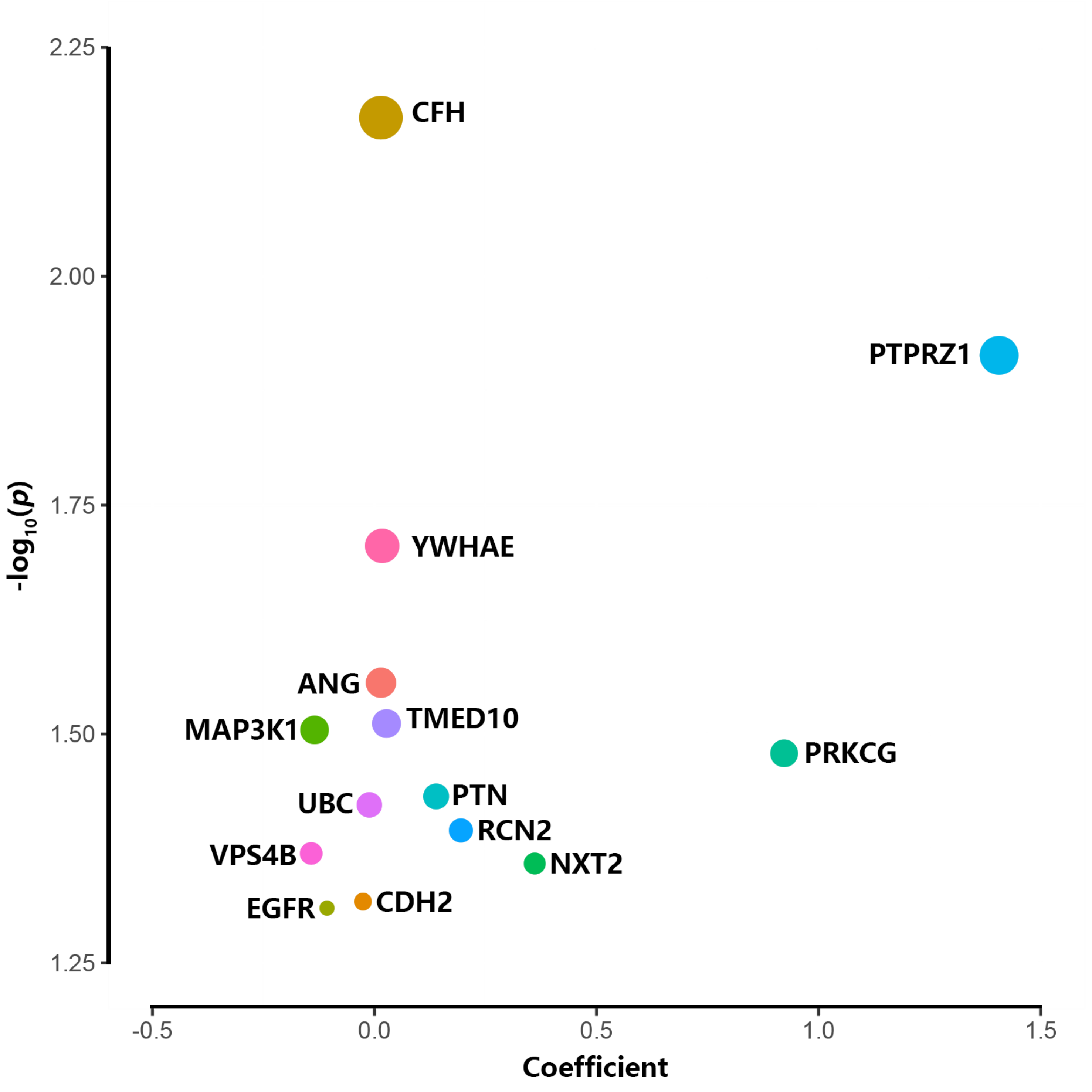

Supplementary Figure S22. Genes within identified multi-omics-altered subnetwork with expression patterns correlated with patients' overall survival in TCGA-CHOL dataset.

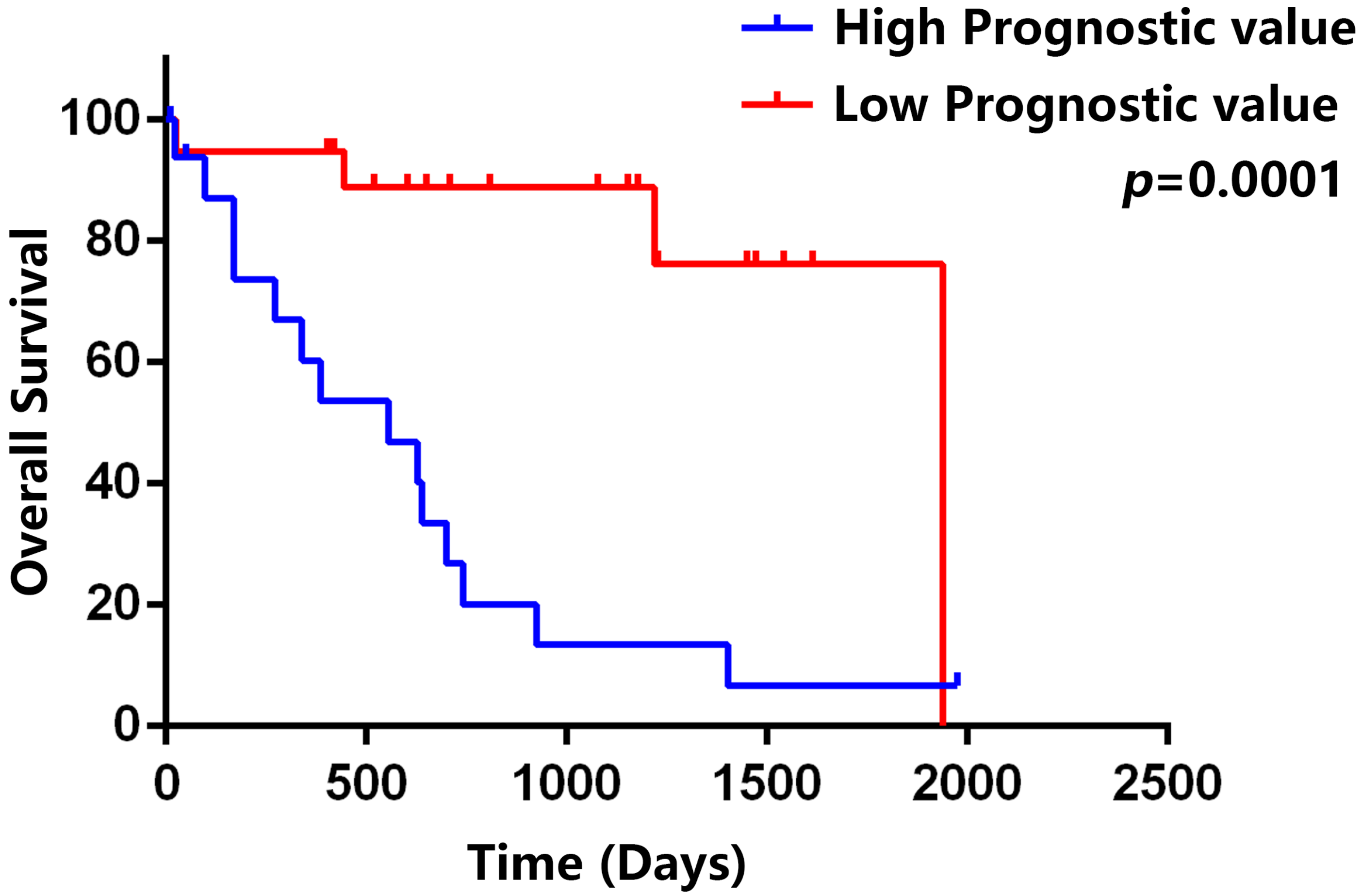

Supplementary Figure S23. Kaplan-Meier analysis of overall survival for CCA patients in TCGA-CHOL datasets using acquired prognostic value.
