# Supplementary material for: Genomic and Transcriptomic Landscape of Tumor Clonal Evolution in Cholangiocarcinoma
Source: Front Genet. 2020 Mar 13;11:195. doi: 10.3389/fgene.2020.00195 (PMC7083074; doi:10.3389/fgene.2020.00195)
Supplement: Supplementary file 2 [file Table_1.DOCX]

# Supplementary Table S1. Multivariate Cox regression analysis of CCA patients in TCGA-CHOL datasets

| Variable | Hazard Ratio (95% CI) | *p* value |
| --- | --- | --- |
| PTPRZ1 | 4.055 (1.177, 13.972) | .027 |
| CFH | 1.028 (1.012, 1.043) | .000 |
| RCN2 | 1.384 (1.107, 1.731) | .004 |
| VPS4B | 0.829 (0.705, 0.975) | .023 |
| Formula: 1.4*PTPRZ1+0.027*CFH+0.325*RCN2-0.187*VPS4B | | |
